# Supplementary material for: Long noncoding RNA PAHAL modulates locust behavioural plasticity through the feedback regulation of dopamine biosynthesis
Source: PLoS Genet. 2020 Apr 29;16(4):e1008771. doi: 10.1371/journal.pgen.1008771 (PMC7241820; doi:10.1371/journal.pgen.1008771)
Supplement: S2 Table — (DOCX) [file pgen.1008771.s002.docx]

# S2 Table. The proteins from *PAHAL* pulldown identified by mass spectrometry.

| Protein ID | Annotation | Gene ID | Score * | Expect |
| --- | --- | --- | --- | --- |
| **13KD** |  |  |  |  |
| J3JYE6 | Histone H2B | LOCMI15324 | 135 | 5.30E-06 |
| H9J2V3 | Small nuclear ribonucleoprotein SmD1 | LOCMI17236 | 62 | 8.90E-06 |
| J3JZ94 | Small ribonucleoprotein particle protein SmD2 | LOCMI17058 | 35 | 0.011 |
| **25KD** |  |  |  |  |
| J3JX90 | ATP synthase subunit beta | LOCMI15919 | 210 | 1.40E-10 |
| Q9NHZ7 | Aminopeptidase 3 | LOCMI12891 | 107 | 5.10E-09 |
| J3JWT8 | High mobility group protein DSP1 | LOCMI06203 | 97 | 7.30E-09 |
| H9JAQ8 | Sarco/endoplasmic reticulum calcium ATPase | LOCMI06569 | 87 | 3.80E-05 |
| B3VBE3 | Vacuolar ATPase B subunit | LOCMI15986 | 27 | 3.40E-03 |
| H9J9M8 | Rab-protein 14 | LOCMI13882 | 29 | 0.033 |
| J3JV53 | Splicing factor, arginine/serine-rich 2 (SRSF2) | LOCMI16788 | 21.8 | 0.011 |
| G1K0S0 | Ubiquitin (Fragment) | LOCMI09522 | 21.5 | 0.055 |

* “Score” indicates ions score that is -10 × log(*P*), where *P* is the probability that the observed match is a random event.

The sequences of these genes are available in the LocustBase (http://www.locustmine.org).
