# Supplementary material for: Long noncoding RNA PAHAL modulates locust behavioural plasticity through the feedback regulation of dopamine biosynthesis
Source: PLoS Genet. 2020 Apr 29;16(4):e1008771. doi: 10.1371/journal.pgen.1008771 (PMC7241820; doi:10.1371/journal.pgen.1008771)
Supplement: S3 Table — (DOCX) [file pgen.1008771.s003.docx]

# S3 Table. Sequences of all primers used in the study.

| Primer | Sequence (5'−3') | Notes |
| --- | --- | --- |
| **Primer for qPCR** |  |  |
| pre-*PAH*1 F | GCTACGCACTTTATCCCTTG | To amplify *PAH* pre-mRNA |
| pre-*PAH*1 R | CTCCCGTCATCAGCATCG |  |
| pre-*PAH*2 F | ATAATAAGATGGCTTCCTGTG | To amplify *PAH* pre-mRNA |
| pre-*PAH*2 R | CGTGGAAACCAAGGAACAG |  |
| *PAH* F | AGTACAGCCTGAGTGGAAAA | To amplify *PAH* |
| *PAH* R | TGTGTGTATGGATTGTAGCG |  |
| *PAHAL* F | TATTGTGAGTCAGGATGGTG | To amplify *PAHAL* |
| *PAHAL* R | ACCTAACAACTTTGGACGAG |  |
| *RP49* F | CGTAAACCGAAGGGAATTGA | To amplify locust *RP49* |
| *RP49* R | GAAGAAACTGCATGGGCAAT |  |
| *β-actin* F | AATTACCATTGGTAACGAGCGATT | To amplify locust *β-actin* |
| *β-actin* R | TGCTTCCATACCCAGGAATGA |  |
| *U6* F | GAACGATACAGAGAAGATTAG | To amplify of locust *U6* |
| *U6* R | AAATGTGGAACGCTTCACG |  |
| *LOCMI15324* F | AATATATCGAAGGGCGACAAG | To amplify *LOCMI15324* |
| *LOCMI15324* R | CGACCGCTTGTTGTAGTGC |  |
| *SmD1* F | TATTACGGGTGTTGATGTTGC | To amplify *SmD1* |
| *SmD1* R | CACCTCTTGCTGCTTCCTTC |  |
| *LOCMI17058* F | GCCGAAATCTGAAATGACGC | To amplify *LOCMI17058* |
| *LOCMI17058* R | CAGCTGCTGTGGCAAGTGG |  |
| *LOCMI15919* F | CTGATGATTTGACTGACCCTG | To amplify *LOCMI15919* |
| *LOCMI15919* R | CTCAAGAGGGACGAGTTTTC |  |
| *LOCMI12891* F | CAGTGCTGTCTCATCTAACAC | To amplify *LOCMI12891* |
| *LOCMI12891* R | GCCCACTGAACATTGATGCG |  |
| *LOCMI06569* F | GCACGAGACGTATGGCTAAG | To amplify *LOCMI06569* |
| *LOCMI06569* R | CATTGAAATCTATGGCCGAATC |  |
| *LOCMI06203* F | GAGCCTGTTGTAATGACTGG | To amplify *LOCMI06203* |
| *LOCMI06203* R | CAACCATTGTCTTCCACCTC |  |
| *LOCMI13882* F | TATGGCGGACTGCCCTCAC | To amplify *LOCMI13882* |
| *LOCMI13882* R | AGTTGCGGGTGTCCGTCAG |  |
| *LOCMI15986* F | GTATTGCCATCACTCAGTCG | To amplify *LOCMI15986* |
| *LOCMI15986* R | GTTTGAGGGTGTCTTGAGTC |  |
| *SRSF2* F | CACTGTCCCACCCCTTTG | To amplify locust *SRSF2* |
| *SRSF2* R | AGTTATCTCATACAGGGGTC |  |
| *LOCMI09522* F | CATCTGGTATTGCGTCTGCG | To amplify *LOCMI09522* |
| *LOCMI09522* R | GTCAGATAAAGTACGCCCATC |  |
| *GAPDH* F | GAAGGGAATCTTGGCATAC | To amplify locust *GAPDH* |
| *GAPDH* R | CACAACACGGTTTGAGTATC |  |
| **siRNA Oligoes** |  |  |
| *GFP* F1 | GTACAACTACAACAGCCAC | To amplify DNA template of si*GFP* |
| *GFP* R1 | GTGGCTGTTGTAGTTGTAC |  |
| si-Lmi-*PAHAL*1 | TGATTGTGTGGAGGTTTAT | Three RNAi Oligoes for *PAHAL* siRNA |
| si-Lmi-*PAHAL*2 | GATTGTGTGGAGGTTTATA |  |
| si-Lmi-*PAHAL*3 | GTATGATTGTGTGGAGGTT |  |
| **The sense-specific PCR primers** | | |
| Primer 9 | AGGTTCTGGGGTGTATAGTG | Primer 9 + Primer 10 for *PAHAL* |
| Primer 10 | TCAATACCTAAGTTCCAAATAC | Primer 9 + Primer 12 for *PAH* |
| Primer 11 | GGACTAGCCACTTGAAAATAC | primer 11 + primer 12 is no PCR product |
| Primer 12 | GACATGGGGAAAGGTGTTC |  |
| Primer 13 | AAGGGAGGCTAGACCAAT | Primer 13 is the specific reverse primer for cDNA synthesis of *PAH* and *PAHAL* |
| **Primer for FISH** |  |  |
| *PAHAL* F1 | CTGCCTGCTTCTTGTCATG | To amplify the DNA template in *PAHAL* RNA probe 1 synthesis |
| *PAHAL* R1 | GACAGGTTGTCTTGCGATC |  |
| *PAHAL* F2 | AAGGTTATACTAGTATCTATTTC | To amplify the DNA template in *PAHAL* RNA probe 2 synthesis |
| *PAHAL* R2 | ACCTTCCATTGATAGAAACAG |  |
| *PAHAL* F3 | CTGCCTGCTTCTTGTCATG | To amplify the DNA template in *PAHAL* RNA probe 3 synthesis |
| *PAHAL* R3 | ACCTTCCATTGATAGAAACAG |  |
| *PAH* F1 | AAGACCCGATGCTGATGACG | To amplify the DNA template in *PAH* RNA probe 1 synthesis |
| *PAH* R1 | GCTTGGTCAACTCGCGGAAC |  |
| *PAH* F2 | ACTATGCCGTCAAGACGGCC | To amplify the DNA template in *PAH* RNA probe 2 synthesis |
| *PAH* R2 | GCGCACTCCAAAAGGTCGTG |  |
| *PAH* F3 | AGGAAGCGGGCAGACGAG | To amplify the DNA template in *PAH* RNA probe 3 synthesis |
| *PAH* R3 | CTGGAAGTAGGCGGAGTTC |  |
| **Primer for dsRNA** |  |  |
| *PAHAL* F | CTGCCTGCTTCTTGTCATG | To amplify the DNA template in ds*PAHAL* synthesis |
| *PAHAL* R | GTAAGGAAGCGGAAACACC |  |
| *SRSF2* F | GTCGGCACAGTCGTCGTAG | To amplify the DNA template in ds*SRSF2* synthesis |
| *SRSF2* R | TCCTTAGCAGCTATCTGGAAG |  |
| *GFP* F | CACAAGTTCAGCGTGTCCG | To amplify the DNA template in ds*GFP* synthesis |
| *GFP* R | GTTCACCTTGATGCCGTTC |  |
| *PAH* F | AGGAAGCGGGCAGACGAG | To amplify the DNA template in ds*PAH* synthesis |
| *PAH* R | CTGGAAGTAGGCGGAGTTC |  |
| **Primer for RACE** |  |  |
| *PAHAL* 3′ RACE | AGGTTACAAAGGACTACCTGT | For *PAHAL* 3′ RACE |
| *PAHAL* 5′ RACE | CTAGTATAACCTTAGTTTTTTGGACA | For *PAHAL* 5′ RACE |
| *PAH* 3′ RACE | GAAAACTGTGGTTATCGTGAG | For *PAH* 3′ RACE |
| *PAH* 5′ RACE | TGAGGCTTTGAATCCAGTATTTC | For *PAH* 5′ RACE |
| *SRSF2* 3′ RACE | CAAGGAGGGTATTAATGCAG | For *SRSF2* 3′ RACE |
| *SRSF2* 5′ RACE | TGCTACGACGACTGTGCC | For *SRSF2* 5′ RACE |
| **Primer for cloning** |  |  |
| *PAHAL* F | GGTAAGCAGCACATTTTGTACA | To amplify full-length *PAHAL* |
| *PAHAL* R | GATGTACTCCATACTGCCGG |  |
| *PAH* F | GAGACAGCGCGGGGCCAG | To amplify full-length *PAH* |
| *PAH* R | CTTTAATTCTAAATGAGCAGGGTA |  |
| *SRSF2* F | ATAGCAACTGACGTCATACG | To amplify full-length *SRSF2* |
| *SRSF2* R | AGTGATTATCTACTGCGTTTG |  |
| **Primer for mapping analysis** | | |
| *PAHAL-*F 1 | GGTAAGCAGCACATTTTGTACA |  |
| *PAHAL-*F 207 | TATTTTCAAGTGGCTAGTCCAC |  |
| *PAHAL-*F 481 | GGCTTTCCGTGTCTTCCAT |  |
| *PAHAL-*F 746 | AGTCACCGGCTAGAAGTAAA |  |
| *PAHAL-*F 1092 | CTGCCTGCTTCTTGTCATG |  |
| *PAHAL-*F 1436 | ATGGGACACTTGACAACTAC |  |
| *PAHAL-*F 1968 | CTTTCTGAAGTAGGGAGCAT |  |
| *PAHAL-*F 2369 | GTGGAGGTTTATAGGATCGG |  |
| *PAHAL-*R 221 | AGCCACTTGAAAATACATCACC |  |
| *PAHAL-*R 495 | AAGACACGGAAAGCCAACC |  |
| *PAHAL-*R 1104 | AAGAAGCAGGCAGGCAAAA |  |
| *PAHAL-*R 1460 | ATGTAGTTGTCAAGTGTCCC |  |
| *PAHAL-*R 1988 | TATGCTCCCTACTTCAGAAAG |  |
| *PAHAL-*R 2369 | CACAATCATACAGGAACAAC |  |
| *PAHAL-*R 2613 | GATGTACTCCATACTGCCGG |  |
| **Primers for vector construction** | | |
| *PAH-*F1 | GCAGACATTTGGGAGTGCGC | To amplify –1,116 to +89 nt of *PAH* with *PAH-*F1 and *PAH* R |
| *PAH-*F2 | GCATTGCGTTTTGCCGATTAC | To amplify –554 to +89 nt of *PAH* with *PAH-*F2 and *PAH* R |
| *PAH-*F3 | CCTAAACTTTCCTGTAGGCTTC | To amplify –364 to +89 nt of *PAH* with *PAH-*F3 and *PAH* R |
| *PAH-*F4 | CAGGTTGCACTACGTCCGTC | To amplify –201 to +89 nt of *PAH* with *PAH-*F4 and *PAH* R |
| *PAH-*F5 | GCGTGCAGGCGCGCTCTC | To amplify –48 to +89 nt of *PAH* with *PAH-*F5 and *PAH* R |
| *PAH* R | TGTGTTGCGTTGCGTGTGTAG |  |
| *Lacz* F | TACTGTCGTCGTCCCCTCAA | To amplify 3000 bp *lacz* |
| *Lacz* R | GCGGATGGTTCGGATAATGC |  |
| *SRSF2* ORF KpnI F | GCGGTACCCTAGCCACCATGAGTTACGGGAGGCC | To amplify the *SRSF2* ORF |
| *SRSF2* ORF XhoI R | GCCTCGAGGCCTTAGATCTTGATCGAGAC |  |
| *PAHAL* KpnI F | GCGGTACCGGTAAGCAGCACATTTTGTACA | To amplify *PAHAL* |
| *PAHAL* XhoI R | GCCTCGAGGATGTACTCCATACTGCCGG |  |
| *Rluc* F | TAATACGACTCACTATAGGATGACTTCGAAAGTTTATGATC | To amplify *Renilla* luciferase containing T7 promoter |
| *Rluc* R | CTCGCTCAACGAACGATT |  |
| *PAHAL* F | TAATACGACTCACTATAGGGGGTAAGCAGCACATTTTGTACA | To amplify *PAHAL* containing T7 promoter |
| *PAHAL* R | GATGTACTCCATACTGCCGG |  |
