# Supplementary material for: Long noncoding RNA PAHAL modulates locust behavioural plasticity through the feedback regulation of dopamine biosynthesis
Source: PLoS Genet. 2020 Apr 29;16(4):e1008771. doi: 10.1371/journal.pgen.1008771 (PMC7241820; doi:10.1371/journal.pgen.1008771)
Supplement: S4 Table — /SI_Caption> (DOCX) [file pgen.1008771.s004.docx]

# S4 Table. The antibody epitopes of PAH and SRSF2.

| Name | Antibody epitopes |
| --- | --- |
| PAH | MSYGRPPPRIDGMISLKVGNLTYRTTPEDLRRVFEKFGTLGDIYIPKDRFSRESRGFAFVRFYDKRDAEDALDAMDGRLLDGRELRVQMARYGRPSSPYRRH |
| SRSF2 | SPRDGDQVGGLAKCLKLFEKHSVNLIHIESRSSQRFPSQYEFMVECAPGGDIGGAVTNLRQNSAYFQIISRNHKDNRDTVPWFPRRIRDLDKFANQILSYGAELDADHPGFTDPVYRARRKYFADIAYNYK |
