# Supplementary material for: Long noncoding RNA PAHAL modulates locust behavioural plasticity through the feedback regulation of dopamine biosynthesis
Source: PLoS Genet. 2020 Apr 29;16(4):e1008771. doi: 10.1371/journal.pgen.1008771 (PMC7241820; doi:10.1371/journal.pgen.1008771)
Supplement: S1 Fig — The red bold text represents the exon sequence of PAH. The bold text with underscored characters represent the full-length sequence of the PAHAL. (PDF) [file pgen.1008771.s005.pdf]

> Full-length sequence of *PAH* locus

GACAGCGCGGGGCCAGAGCAGGTGGCGAGCCGAGGAAGCGGGCAGACGAGAT
CGGCAGGCGCAGCGACTACACACGCAACGCAACACAATGGGACTCTCTGAGCTG
CCGCCGTCGCCCGAAGAGATCGTCGAAGACGTAAGTTAATACTTCACCAAGTGCT
AGGCCGACCAACACGTGATCGTAATTTTAGGGAGATGGCCGACCGTGATTTTAACCC
TTTAATTCATGACTTTTTATTTGAAGCTAAAAAATACCACGATTAATGTATAGCGCGT
ATATACTGAGAAAAATTTTGAAAAAAAAATCCTTAAAATTATTATGCACCACTAGCA
ACAAAAAGTTTATAACTGGTCAGCTATACAGTGCTTCTGTGCTGTACCCGATGTAGTA
AATGTCACTATTATGCCTCTCATATTTCCGGAAAGTAAGTCATAATGCAACAGCAGTA
TGTGATTGACTACTTTTACATGGCAAAGTAGGAATACTATTCTAAACAAACGAACATG
TTAGGAGTAACTCAATGCAACTCACTGTCGAAATGCTTCAGTAACAGTCTGCAATTTA
TAATTTATAGGACGAGCTACTAGCATGTATAACACTATGTATACAAGCAGCTATAAAT
GAATTTGTGTTAAAATTTTGAACAGTTTGAGAGATAATGGGAAAAATTAAGTATGTAC
CTGATCAGCTACACTATGCATTGAAGGGTTAAGGAAGGGGTGACATAATCGCACCAA
AAAAGGAGGAACAGGGGTAGCCTAATGACGCCACCTTAACGGAAACCGGAATGTTTT
CGCCGTATTAAAAGTGTATCTTTTTCTTACAAAATAATACTATTCTTGTTATGGTGGTT
GTCTTCAGTCGAAAGACTGGTTTCTTGCGGCTCTCTACACTACCCTTTCCTGCACAAA
AACCTTTATCTCCGAATAAGAAAGGAAACCCACATCCTTTTGAACTTCGCCACTTACC
TTCTCTCTTTGCGATTTTTACACCAATGCTAAATTGATAATTCTTTGATATCTCAGAAT
GTATCGCACCAAGCTATCACTTCTTGTAGTCAAGATATGTCTTAAATTTCTTTCCTCCC
CCGTATTTTGTTCAGTGTCTCCTCATTAGTTACTCGATATAATCGTCTAATCTTCATAA
TTCTACTGTAGCACCCCATCTTTTTCTCTGAACTCATCGTCCACGTTTCACTCCTGATC
ATTTCCGACGGAACGTGTGGTGCAGTGGTTAGCATCACAGATCGCTGTGTTGTGGTCC
CCAGTTCAAATCCTACCACTGGCAGAAGTAATTATTTGTTTGTATTTATTATTTCTCGA
AGTTCTACATGTTTGTCATGTTTGATGTACTTGTAAGTTCCAGAAAATTCTTTATATGT
ATAAACAGTGGAATACTCCAAGCAGGAGTCAGTCTGTAGTGAGGATACGCTTGTGTT
AGCTATAGACGTGATCTAAGTGTAAAGTGTTGTGCTATGTTAACCACCAGCCTTCGAA
TTCTAGACGAGCTGCAGTTTGCAGCGTGATAATATTACACTCCAGACAAACACTTTCA

GAAAGGACTTCCTAACATTTAAATTAATATTTGACGTCAACAAATTTCTCTCCTTCGA
CAATGCTTTTCTTCGCATAGCCATTCTCTATTCTATATCTTCTTTATTTCGACCACCAG
AAGTTATTTTATTATGCAAATAACAAAACTCGTCTACTTCTTTTATTGTCTCGTTTCCT
AATCTGATTCCTTCATAATCGACTAATTTTTCTTAGAATTCATCCCATTCCCTTTGTTTT
ACACTTGTTAATGTTCGTCTGATATCCTACTTTGAAGACACTATCCATTCCATAATACT
GTAGCTTCACCGATTATTACAACTTTGTTATAATTGTGTTCAGCCCTCTGCCTTTCTTTT
TTTTGCGTTTTATTGTTAAAGATACGTCGTGAAAGTAGCTCTAGTGGCGCTGTTACCCT
GGTTTCTGGTATAATAACGCGTTTTCCATGGGGTAAATACAGCATACTTTTAAAAATT
CAAAAAAGTAGCCTGCATATTGACAGACTACTAGTAAAAAAGTATTGCAACTGTTGT
AATTTAGACAAGTGAAAAGGTGGGGGTTAACAGCGTTGATATATTTGTAGGATGACA
TTATAATTGACCACAGCTGTTTATTTAATGACCCAATGTTACCGGTTTCGGTTTTACAT
CATTTTCAAGCGCCAGCAGAGCTTAAACTTTTGATTAAAATGACATGTTACAATCTTG
AAAATGAAAAAGTATAAAATAGATATCTCGCATTCTACTGGAATTGCTAGATGTGTAT
CTCAAGAGACTGCTCATTACTTGTAATTTACGCACATCATTCTTTTCAGTGTGACAGT
AAGAAGGCGTAATTATACTTTCGACAGTGAGACAGTAAGAAGGCGTAATTATACTTT
CGATTAAAACTGGAATAAAGATAAATGTTATAAGTTATTCCTGTGATATCTTAAATAT
AGTTTTAACTCTACATATTTGCCATAATATCTACAGCTGTACCACAGATCATTTTGTCC
ACATTCTCCACAAATTTCCACACTGCTTTACGCACTCTTTCATAAGATTTTGGAAAGA
GATTCAGTAATTCAGAAGGTTCATGCGCAATCTCACCTCGTTTACATTGTTCCTGCATT
TGAACTCACCTCACCTTACCAGAGATTGACAAAACAAACACTACGGTGCAGCTGTCG
CAAATCAGTAAGAGACTTGAGAACATAAATAATTTACTAATGAGCTACGCACTTTAT
CCCTTGTATTACGCCAAACAGGGCATGATTCTGTAGAGAAAGTAATTCCATGCCAAA
CCTTTTGAGTTTGTTCAAAGTCGTCACCCGTCGACTGCTTGTCATTACTTTCCCGGTCT
CGTGTAGGCGATGTAATTCTAAGCTTGCGGCGTGCTTTCTGTTACAG**CCGATGCTGA**
TGACGGGAGGCAACTACATCAAGGAGGGCCTCGACTCAGCCAAGAGCATTTGCC
TCATCTTCTCGCCGCGAGACGGGGACCAGGTCGGCGGACTCGCCAAATGCCTCA
AGCTCTTCGAGGTGAGTCACTCTCCAGTTCTTCGTTATTCATATAATTATCCTGGGTT
GGTGTCCACATAGTATTGTAAACCTAACAACTGGTTCAATTACTATTACAAGTGATCT

57 TCATTGGGTTTGTCACATAATCTAAATATATATACACGAATGTTCGTCTGTATGTCCTC

58 TATGTGTTCATAAACCATTCATTCGTTTGCAATGAAACTTTGGTGAACTGTTCACCGTG

59 CGCCCACGAAGATTCCTGATTCAAAAACAAGAGAGCACGACACATAGCTCAGGAGAT

60 ATGACGTCATAAATAATGAGAGGCCACCGCGAGAGAATACGCAGATTTATGCCTCAA

61 AATTGGAAAATGAGAGTAGTTAGGGACCTGCAACAAGGTTTGCATGTAATTTCGGAC

62 GTTTATGAAGCGTTTTATAACCGACACCACACACACACACACACACACACACACAAT

63 GATGAAACGATAAGAAGTCTACCGCTCACAACAGTTTCGCTGTAGATGCTGTAAAAT

64 TGTCGCATCAAATAAAGTTAGTATTACAGATTTGCATGTTACTATTTCTTTTTGTCTGC

65 AAGTCTCCCTTAATTCCCTTGCATCCAAAATTAAGCAATCTGTTTTCTTAATCTCCATC

66 TATAGAAAAGCGAAAGGACACTCACTCACTGACTAAACACGAAATCTAGAAAACTAC

67 TTCACCTATGAAGCTGGAATTTGGCAGGAATGTAGTTTATAGGTAGAAAAATCAGCT

68 AAGAAAGTATTTTGTGATAGGTGCATGGCGGAGCATACTTCTCCTACAGACTCAAATC

69 TACAGTTTCATTCCGATCTTCACGAAATTCCGCACACTTGATCTTCAAAACAAGGGGT

70 AGATCACCTTCTGCCTTAAGTTTCATACGGTGCATGGAGGAGGGTACTTTGCAACATA

71 AAAATGGATCCCTAAATACCTTAACTTTAGAACGGTTCGAGCGATTTCGCAGATACTT

72 TTTTTTTTTTTTTTTTTTTTTTTTTTTTTTTTNNAACAAGGGTTGTATGAAATAAACCTG

73 GAATATTTGAGAACAACAAAGTGCGTTTTTCAGTGTGACCCTGAGTTTCTATTGCATG

74 GAAATCTAAAGCTTTGTTTCGATATTCCTGAAATTTTGATCACTCCACCCTTTTTTTTT

75 AAAAAAATAAATAAATAAAAATAAAAAAAAACTAGTGGAATGCCACTGTCTGTATAA

76 AATTTACTTAACTTACGGAATTACACAGAGTGCATATTTTTGCTCCAATCTTTATGAAT

77 TTTTGCATACATGACCTTTAAACCAACAGGAAGATCACTGGGTATATAAATGTTTGTA

78 TGGTACATGACAGAGGGTACGTTGCAGAAAACTTTGCCGAGAGAGAGAAAGGTGGA

79 AACGAACAGAGAGAAGGGAACTGGACAAATTGAAGGAACCAGAGTTGGTTGAGGAT

80 TTCAGAGGGAGCGGGGAGGGGGGTGTAGGATAAGTAAGCACTCGGGCAAAACCGGG

81 TTTACATGTAAAACTTCTCTAAATTGCTTTACACCAACCTATTACGCGGCAGTGAAAC

82 TGAGAAGTTGCTTACGACTAACTTGAAAGCTCTGCGGAGACACACACGACAGATATG

83 TCAAAAGCCTTCACTTAAGCTTCTTTGCTTTCTTTGTCCCTACGCAACAGAATGTCATC

84 GACCTTCGAGACAATGCCAGTGATACTTACCGCTCACATGAAAACGGCTTATGAGTA

GGATACATAAATTGAATAGCTAATATTTTGTTTACCTATCTTTCCCCATTACGGAATTG
GAGGTTTCTAAGGCGTCTGCAATAGCTACGATAACTTATACTAAATTAGACTTACTCT
TCTTTATCAGTCACCACTTAAAGAAAAGATTAGCATAATGTATGCTTGTTGTTCTGGT
GTTATATTAGCGATTGACACCTTAGTAGCAATAGCCAGTCACTCTTCCGCCATTCTGC
TAAATCTCCACTGTGTTATATCGGCACGCGAAAACATGTTTGCTTACTTTCACTCTGTC
AGGTTCTACGGGTCAGTTGTAGTTGTGTGCTTTCCTTGGACAGTGAGGGCTAATAGAA
CGTCTGCACTGTAATTTGGTATACTTTGTATAAGACACTGTCCAAGGGTCTAGTAGCA
ATTATTCCACTGCTATCTCAGTGCATATACTTTCTCATGAGGCTTTCACTTATTAACAA
TGTCTCAACTGATAGTAACGTTACATGCACTACGCGAACAGTAGACGAAAAAAAAAG
CACTTATACGACTGCTTCTTAACAATTGTTCGGATCAGTGTACACTACTATGCAAGAT
TCATTTACAACGACACTCCAACTTAAGTACAAAGAGAGCGATATAAACAGGGTCGAT
GTGTTTGTTACACATTTCCTACATTATTCCAGGCTTTCTTTCTATAGTATAAAACATTC
CGGTGTCATGCGGTATCTTATACATTTTCTCTAACAAATATATCTCTTTTAAATCAATA
TTCCTTGTACGCCTGTATACAGGGTGACTCACTTAAACTTTTCATCCCAAATACCTCTG
AAACAACAATGGATATTGAAAGACGACTTCCGACCATGAACGTACGGCAGGGATAAT
GAAAGTAAGTACTATGAAACATTCTAAAACGTGTAGAAATATTATTTTCAAAGCAAA
TATATGTGTTCTTAAATGGACACCGCATATTATTTTTCATGTAGTCAATAGCATGAAT
GGTGGTGGTCGCATCGCAATACGTCATTTACGTGCCGAGAAATTGGGAAGCGAATTT
GAGTCTTCAAATAAATGATGCGCGTAGACAGCACAGCGCACCATTAAGGTGAGGTTC
ACGATTGAGTTTCGGGACGTGCGGTAGCTGAGCGTCTCGTTTATTTCAAGCGTCAAAT
TACAATTTCTCGGGATGTAACTGACGTATTGGGATGGCGCCGCCCGCTTTTCTGATTT
CTGGCTTTGCGCAGAGATTTACTACAGAGTAGTATTAACCATTACGGCTTGGCCTACT
AATTCGAGTTATTATTGTGTGTGGGATTTGGACCACTGTATTGAATCGTGTCTGCTGGT
TCAGGGAAAGTGGATGGTGAATAATTCTGGGTTACCCTATGTTGTAGACAACAGAGT
AACCGACCGCTTATAGGGGTCGTCTGGTTCGTTTGGTTGACAAAGTTGCCTGGTTAGA
GGATTCGTGGAATCCCGCGTGCGTGCATTGAACTTTCGTGCTATTTTTTGGAACCTCTC
CAGGATGTCTTTCTTTTGTGCAGCTTCTTGTAGGTACTGGTGTGAGAAGTCAGACGCT
ACGTGAAACACACGGCGAAGTATCTTATTTTGTTAACTTTGCTGTCTCCTCATGGGGT

GTCCATTAAAAAAATATATTTAAAAAAAATTAAAAAACATAAGTTTGTGTTGAAAAT
AATTTTTGTTTACTTTCTAGAATGTCTCATAGTATTTGCTTTCATAATTTCGAAGTCTGT
TAGTTCCCGTCGTGCCGCCACAATGAAGTCAGAAATCTTTCCATGTGGTTTAGCAGGA
TACAAATGACGGCTTTTACACCTTACGGACGCGATATTACCGCAGTCTGTATCTGACA
AGGAGAGGACGCCTACTCGTCGTCGCCGAATTCGTCCAAATTAATGATACATGTAGG
GCATGGGGAGATATGAAAGTGCACCAAGTGAGAACTCCAGATGGCTAAGCGTTTAGG
AAATAAAAGGAAGTGTGATACGGATCTATCACCATATGCATCTTATCAACTGTCCCTG
TGACGGTGTTTGGCTCAGCGGTAAGAGATCGAATTCGAGTTGAGAAGGTCACGGGCT
TAACTCCACCCCGCGGCAATTTTTTTCTGCTCCTTGTCAATGCGTACACCTACATATTG
TTTCCGTAGGGATCGTGAGTACAAGATTAGATTAATTACAGCACGCACGGAGGCATT
TAAACAATCATTCTTCCCACGCTCTATACGTGAATTGAATGGGGAAAACCCTAATAAC
TGGTACAGCGGGACATATCCTCTGCCATGCACTTCACAGTGGTTTGCAGAGTATTGAT
GTAGATGTAAACTGACGTCACAATTAACCGCAACATTTTTTTCTGATGAATCGAGGTT
CTGTTTACAGCATCATGATGGTCGCACCCGTGTTTGGCGACATCGCGGTGAACGCACA
TGGGAAGCGGATTCGTCATAGCCATACTGGCGTATCACCCGGCTTGATGGTATGGGG
TGCCATTGGTTACAAGTCTCGGTCACCTCTTGTTCGCATCGACGGCACTTTGAACAGT
CGCCGTTACAGTTCAGATGTGTTACGACCCGTGGCTCTACCCTTCATTCGATCCCTGC
GAAACCCTACATTCACTTCATCAGGATAATGCACGACCGCATATTGCAGGTCCAGTAC
GGGCCTTTCTGGAATACGGAAATGTTCGACTGCTGCCCTAGCCAGCACATTCTCCAGA
TCAATTGAAAACGTCTGGTCAATGGTGGCCGAGCAACTGGCTCGCCACAATACGCCA
GTCACTGCTCTTAATGAACTGTGGTATCGTGTTGAAGATGCATGGGCATCTAAACCTG
TACAAACCATCCACGTTCTGTTTGACTCAATGCCGTTATTACGGCCAGAGTTGGCTGA
TCTGGGTACTGATTTCTCAGAATCTATGGACCCAAATTGCGTGAAAATGTAATCACAT
GTCTGTTCTAGTGTAATATATATACGTCCAGTGAATACCCGTTTATCATCCTCATTTCT
TCTTGGTGTAGCAATTTTAATGGCGAGTAGTGTAGATTGGTGTGGCGTCTGTTCTTTCG
AACAAGTCCGAAGAGGGCGTTTGTTTCATTTACTTCAGCGCAAAGACCTCGCACGGG
AATCCATAATGGGAAGAGTAAGGCTGGTTGCACACACACCGAGTTTGGACGGCCGGT
AAATATCGGGCGGTATTTTACCGGTGGAGCGCTTCTTGCACAGACGCCGGCATTTCAC

CGGTCAAACGAGCAGTCGCTTGGCTGTCGTGGTCGACTGAAGCCCAAGAGGGCATGG
ACAATGGTGGAAGTTTGAGACGGACGTTAGGCGACGCGGGTAGCCAACTGGTGCCGG
CGTTCTCTGTAATAAAAAAACTGAGCTAAAAACTCTACCAACAACCCAGAACGAACA
CGAACAATCGACCGCAAAGAATCGATTTACCTGAGGGGGAAAAAATCGTTAAGGCG
ACCGCTTGCTCGAAGCGACAGACCCGGGCTCGATTACCGACACGACTCACGTTTTCAC
TCGTCGTCATTGTATAATGTCAGTGCCCCAAAGCTAAAAGTTAATCCTTTCCTTCCTA
ATCCTTCTTCCTTCCCCTCCCTAATAAATAATTTGTAAAGGTAATAATTTCATAGCAGT
AGCAATTAGAAATACTAAAGCAATTAGCACAGAAATACAAATAATTTGACGTTTTCG
TTGTGGGTTTAAGCCAAATGAGAAGTCCAAGCTCCAAGCGCAGCTCCAGTAATACTG
AAAGGGGAACGGATGTGAAATATGATAATTTACTGCTATATTTCTTCTGTAAATCTGT
GCTATTTGGAAGTTATTGAAGTTATTGAAGCACTGATAACAAAGGCCACTTGGCAAA
GGTTTATTATTTTTCGATTTTTTAAAAATTACTTTATATTCATACTTACATATTTCGATG
AAATTTTTCGTAAATAGAAATATATTACTCAGTCATATGGGCTTAATATTCTTTGAAG
ACTGATTAATATACGTAAACCTCTGGCAAAGAATACAAAGACAATGAAACGTTCATT
TTTTCCGAACTATAAAGACAGGAGATGGGTCTCCATTTACTTCTTGTTCGAACATTGA
GACAGTAAAGTTGTAAATGATTTGAGCCCTGTAGTTATTACGATTTTTCCAGAATATG
TACAATCTATGTAAGTTATTTAAGTTTTATGTAGATATGCTACTTTTTAATTCAATTAT
AAGCATTTTAGTTTAACCGAACTTTTAATGTGTTCTCATTTAGTAGAAAACTGATTCCT
TAATTTTTTTCCTTCTCACATTTTATTAACTGTGATGCAATTTACTTTTCAGGACAACG
AATTCCGCTATTACATCCAATTACGAAATTAATTGTTGGAGGTGTGATCAGAATAGTA
ATCATTTTCATTCCATTCTTGTACTAAATAATTGTTCTGAATACGATCCTGAGATTTTG
TGCCGAGTTATTAAATTTCAGTTTTAAAAATCTGAGCTACCCGTGGACTTTGTTAGTA
AATGATAGTAAATGACGTCTCCAGCAGATAGGTTTATGGCGGTTTTCCATCAACAAA
AGGTTTTTAATTTATTTCTTAGTGCGTATCTCGCATTTTCTTTAAAAATGCTGGATTTTT
TCAATCTTTTAAAAATAGAGGCACAGTTTCATGACTCACATAAATGCTACGACACCAA
TTTGAAATATTTTTCCCGTTCAATCATTATAGCACCGTTAGTGATAGCTTTTTGAAAAA
TATAACCTGGCTTTTACAGTGATTTCGTTCAAATCAATATTTCCTTCAATGTTTTCATT
TATTAGAATCTCAGAACTGTTTCATTTGCAGGAGCTACTCCACTTCAAGAATATTTGT

AAACATATATCCAATATATAAAGCAGAATGACCGTATGTTTGTGTGTACGTGTTGCAT
GAAAATCTATAGTTTTGTTCCAATCTTTATCAAATTTTGTACACGCAAACTTCAATCCA
TGACGACGGTCGCCGTAAGTGTAAGACATATGACGTCATAAACAATGTGATGCGCAA
AAACCTGCTGCATTTAGCGACCATGCTGCTCATTTTCTCCTAAACCACTGGGCCGATT
TCAACCAAATTTGGTACACACATTCCTTACCGTGAGGTAACAATTGCTGCAGGGTTAA
GAATCACATACAACACATGGTTCAGAACTTATGACGTCATAAATATAGGAATCGCAA
AAAAGTGTCACATTATGCGGCGTGACTTTATAATTACGCTAAGAATGAGAGTACTTTG
CGACTTGCAACAAACTTTAAACATTAATTCAAACCTTTCTTAACCTTTTCTCACTGACA
ACCATAGCAACCATAGCAAAATGGACTATAACTACCGCATTCGGTATGACGTTACAA
AGTATAATTTGCACACTACAAATTGTAGCCGCAGGAAATTTTACAAACAGTATACAA
GCATATAGTTGAGTGTACCTTCTAAATTGTATCACAGTACGATACGTAGTTCATGATA
TATGAGTACATAAACAGTAGCGTTCATAGAAAATCGCCCAATCACGCACCAATTTTTA
ATCTATTACTTCTTTACTACTAATTGTATTCCCAAGAGAATTTACATAAAGCTCTTGTA
CGAATGAATATATACCTGAAAAAAGGCAGGAATAAATAAATAAATACCCGGGCAAC
GCTGGGATTTAGCTAGTTAATTAAATAATAGTCATACCTAAAAATTTTAACAATAGGG
GAGAAATGTTTCATTTTGGCGCCCAGCTTGGGGCTCGAACCCACGCTCTGAGATAAAT
AGTCTCATACTCACCGACTGAGTCGGGCCTTCCCAGTCCCCTGCCGAGAATCGAACCC
GGCCTCCCTGCGTCTCGTGGACGACGAAGCAGTAGATATGTAGCGCCAATTCGCTTTG
TTCGAGGAAGAAAAATGAATTATTCCGGTATTTACCTTGAGTGGTTAAAGAAAAATC
ACGGGAAACCTTAACGAGGGTAATACGGCGAAGATTTAAACTACTATTTTCCCGAGC
GCATGACCAGCCTATTAATCACTGCTTTTCCTAGCTCGGTTCTGCAAAGAATTCTACT
GTCTTAACTACTGCACCGCCTTCACGAACGTGTTTCAATGAGAAGCATGACAACAGTT
CACGACTGCGCAGATATCCCCGGAAGGCTGTCCAGAGTGACGCACACATGCGTTGCA
ATAGGACTGTGACTTTCTCCGACTGCCAGAGATTCCAACAAGAGAGCGCTCACGCAG
GGAAAACTTGGGTTTTCACGGCGTGGTTGTTCTTCCTGGTGTGCGCACAGACTGGAGA
CCCCATAACGCCAGCTAATTGCTCTCGTTCAGTGGCGTAACTATAGAGGGGGGGATG
AGGGGGATAGAACCCCCCCTCCCAGAAACCGGAAAAGAAAGAAAGAAAGAAAGAA
AGAAATCGTGCCGGTCGCGGTGGCCGAGGGGACTGAGGCGCTCCAGTCACGGTCTGC

197 ACGGCCACTACGGTCGGGGGTTCGAATCCCCTCTCGGGCATGGATATTTGTGCCGTCC

198 TTAGTATAAGGTAGTTGATAAGTAGATTGTAAGTTTAGGGACTGATGACCCTGTAGTT

199 TAGTCCCATAAGCTTAAAAAAAAAAATAAAAAAATCGTATAACAGCAGTATAGATAC

200 TCTGCAAATCACATTAAAGTGCCTGGCAGAGGGTTCATTACCCACTTTCATAACTTTT

201 CTCTACCGTTCCATTCTCTAATAGCGCGCGGGAAGAAAGAACACCTAAATCGTTCCGT

202 TTTCGATCTGATTTCTCTTATTTTATTGGGATGGCCATGTCTCCCTAAGTAGGTGGGTG

203 CTAGCAAAATATTTTCACATTCAGAACTAAAAGTCGATGATTGAAACTTCGCAAACA

204 GATCACGCCGCAATGAATAACGTCTTTCTTTTAATGATTTCCACCCCAATTCGTGCAA

205 CATTTCCGTGACGCTTTCCCCCCCGTAACGTGATAAAACAAAACGAGCTGCCCTTCTC

206 TGAACCTTCTCAATATCTTCCGTCAGTCCCACCTGGTGAGGGCTTTGGATGGGAGTGA

207 ATTAGTTTCTTCAACGGGAGTGGTAGATAGTAGTCCGTCTGGAAAGTGACTAAAATTT

208 TGCTCGTCAAGCAGATCTATCCTCCCCCCCCAAAAGCCAAGTCCTAGTTACGCCACTG

209 TTCTCGTTGCTATAGGATTGACTGTACCTAGCAGCAACGGCACGTTGAATAGATTGTA

210 AACATAGTTTACAGAAGGCGTGATGTAGTTTTACTAAATACGTCATCTCCTAACAAAC

211 TTTACAGCCGATGTGCCAATTTCCAGCGGGAGCTCACATTCCGGAAAATCTACTGCAG

212 AACTGAATCCTCTATTGTATATTTATTTCCTCAACCTCTTCAATGATTCTTATATCGTG

213 TGCTATCAAGTTTAGAATATGAATTTCGAGAGGCTATGCATACTCCTAATTCCTAATA

214 ATCACTGAGATAACTTTATGGTCCTGTTAGAAAGATCGCCGCAATATTGTATTTAAAT

215 CTACTACTTAAAAACGATCAATGTAACATTCTTTACTTGTTGACAAATCCACTTCAAA

216 ATACGCCCCTCCACGTTTCTTACACCGTTCCATACGAATTTCCTATTGCTCATACTGCC

217 AAATTTGAAGTTATCTTCTGAAACCATCTCCATTGTCTCCGTCACTTTTTTTCACGGAT

218 TCAGCTGTTTAAAAACGTCCTCCCTTGAGTGCAGACTTCACTTTCGGTAACTGAAAAA

219 AGTCATGCAGAGCAAGATATTCGAATAGGGTGGATGTTCCAGAACAGGTATCCCATG

220 CATACTCAAAACCGACTTGAGAGGCGTTCGGATCTTTGTAATTATTTTTTAATAAACA

221 TTCAGAAGTGAAAATGTGAATTTAATATCTACCTGATTTTTTTCCTATTTTTGACAATT

222 CTTTCATTACCGGCATCCACATGAATACTTCGGTTTCTTCAGTGTCTTCCACCGTTGTT

223 GACGTTGAAGGCGCGCTAAACTAAATTAATTCATCGTCAGTCTCTTCTAGTACTTCTT

224 TAAACTGTGAAAACCACTCACTGTCTCTAATGACCAAGCTGTCGACAGGACGTTAAA

GCTTCCTTTCTTTTTTTGCTTAAACCATTCAAAAACTTCATGTTAGTAATTCATCACATCATTGTTTCAATAAATAATGGGCATCTGTTGCTATCTTTCACATTTTCATACAAAATATCTCATTATTTGTTGCTCTTACGTAGTTTTTACCCTATGGTCTGCCGACTTCTCAGTCGGACCGTGGTCTTTGTGTATGTGAAGGTTTCTCCTTGAGGGCCGCGCTGTAGTAGGACCTCATGTTTTCTGCAAGCCTATGACGTCTGGCAAAGGGAAAACTGGCAGCTGCTTAACCTCTGTGTGTGCTCCATCTCTTTAACTTCACTTTTATAGTCTTCTCGTGAGGCTTCAGTTGAAGGAAGTACTTGAGGTCTTCTCGTGAGTTATAAATTGATGAAAGTAATTTACTGGTTGGCTCTTCTGGAAATGTAAGCTCTCGAGATTTTGAAAATAATCAATGACGACTTTGTTCCTTCAAACTGTTATTCCATGCTCTCATTTGTGTGTAACTGTGATAATTAGTCGACTCAGTGGCACTATTGATGTGAAGTCATAATTATGTAATAAAATTTCACAATAAGGTTATTTATTATAGACTGACAAATATACTTTCAGACAGTCTCTTCTATTAACATCGGGCGACGATGACGCAAGCGAGTAGATACTTCAACCTATCATGCAACTCAGCAAAGCAAAACAATATTTGCTCTACAATCGCGTTAGAAACGTCGCAATAAATCAGTCCACATGGGTAGATTACTCAATTTCACGAGATATGGGTGAAGAAAAAAAAAAGTAAGTAAAACTCTTTCAGTTAATGCGACAGTTTGCGTCTCGGGGTGTACCGGTATTCTTTATTAGGACCAGTTCTTACTATCACAGCTAATATCAGCTTATTTCGCTGTTTTTGAGTATTTAATCAAGGTAAGGTGATGGAATCATAGAAATGACAGCGAAGTCACTCGATGTGCTACCATTCTTGGTTCTTTTAAAACTTAAAACTATGCTACAAAATTTTAAACCTGTCTTTATCTATTTTGGTTTTAAGTGTACTGACTGTTTTAAACCATCTATGGAGATTTCACTACCTTAAAAGGCAGCGACTGTTTGTAAACTATCGTACCGGAACTGGAACATTCCTAACGTACGAGGGCGTGTTGGAAGGTTTATGTGAAAACTCTTCAGGCATTTTCTGCGTGCCTATTTCTTTCCCAACGTAGTCATCCTAATAAAGAATCACAGTTCTCCCAACGAGCGATCGGTTTGTTGATACCGTCACTGCAGAAAGTCCGTTGACGGAGCCGTCTCACCTTTGCTTGCAATGCTTCATCACTATCAACGTGAAGTCCTCGAAGGTTTTCTCAGAGTGTTATGTAGGCCTAGTCCGGATGGGGAATGATCCCCGACAGTGGACCCAAGGCTTCTGCTTGCTGCAGATTTCGTAGCGCTCGTGAGTAGTCTGACATTGTCATGTTGAAGGAGAGGATGCTTCATGTGCCGACAAACTCTTTGAATTCGTACTTTTAGTTTTCTGGGAGCCAGAAACTCGTTTACAGCACGCCGTTTCTCACATATCGATATACTTACGTTCAGTGGCGAGGACTGGATTTTGATCTGACGTGCCTTTATTCCT

AGAACCGTTTTAGACTGGTACCACGTCTCTCGCCTCAGGTGTATCGCATAATAAGGCA
TAATATCAGACATTTGTACAAAATGGCGAAGTACTTTTGACAAGCTAGACTGTACCCT
TAGGTTTCTGCCTGACCACATCCTTTAAAATCAAGACATATTGGTAAAAAAAAAAAA
TAGTAAAGTCCTTTTGTGAATCAGAATAGACATCTTAAGTGTAGCTTATCAAAAGTAC
TTTGCCGTTTTCTACGAATTTAAGATATTAGGAAGGATGGAGGAAGGAAGCTTAGGG
TTTAACGTCCCATCGCCAGCTTGGTCATTAGAGACGGAGCTAACGCTCGGAGTTTTAG
GTAAGGATGGGGAAGGAAATCGGGCGTGCCCTTTGAAAGGAACCATCCCAGCATTTG
CCTGGAGTGATTTAGGAAAAACACGGAAAAGCTAAATCTGGATGACCGGAGGTGGAT
TTGAATCCCCATCCTCCCGTATGTGATATTAGGCCGCACTACACGATACATATGAGGC
GAGAGGCGCAGTACCACTCGAAAACGTTTTTACAAATAAAGGCTCGTCTTGGTCATG
GGGGCTGAGTATCTACATGCCCCAAATTACACTTGAAAAAAGTGCAGTTTCGAATTA
CGCATCACCATATATTAATAAAACTTCATTATTTCTCTCTCTTTCAGTTCTCTTCTTTAT
ATATGTATATATAAGAAAAAAAAATGTGCAGGTTAACAAGAATCAACAGCACGCGGCG
AGGGGGTGGGGAGGGGGCAGACTGCGGTCGTTATGCATTGCCATGTTATTACATGCT
ACAATTTGGAGCGGCAGAAGTACTCCATTTGCGCCAGAGAAGCGAAAAAGTAGGCG
AGTAATATACATGACGTCAAATACCTCAACCGATAAAAAGTCATAATTTTTCAGCCCG
CCCTTTAAGGTCAAGGTTGGCATACGTCCTGGATTTCAAAAGACAGTCCTGTAGTTTA
AGAAAGTGCCATGTGTCCCGCTAATACCTGTTCTGGGACGCGATTTATCTCGTATTTT
CTCGATTTATCAGTATCGGAAAAAGAATTATTAAGTTACAAGATCACCCGCTAAGGT
AATATGATGTTCCATCAAAATTGCCCCAGTCGTCTTAGGGACTATAGGGAAAATACAT
GCATACATGGATCTTTGTACATACAGCTGCAAGTCACCTTTTTTTTAAATTATTTTTTG
ACAGTTTTGTTTGTAAAACATAACATACAATCACTTATAGTGCTTTTCATAAAAGTAA
TAACGTTGTCAGCAAGGCATAGGCTTTATATTTGGATTGGTCATTGATGAAAGAAAGT
AGACCCTCCACCAGTTCACTGTATTCAACAAACAGCTACAGCAGTGGCGTAACTAGG
ACTTGGCTTGGGGGGGGGGGGGGATAGATCTGCTTGACGAGCAAAATTTTAGTCAGC
TCCCAGACAGACTACCATCTAACACTCCTGTTGAAGAAACTAATTCACTCCCATCCAA
TGAAAGCTTATGGTAAAACAGCCCAAGTTTCCAATCTTTACCTTTACCATATTGCTGT
TATACGATTTCTTTTTCTATCTCTCTCCCTCTCTCTCTCTTCTTCTTCTTCTTTTCGGGTT

CTGGGGAGGGGGGGGGTTCTATCCCCCTCATCCCCCCATAGTTACGCCACTGTGCTAC
GGAGTACAAGGGCTTAGGCCTACGTAGGATAACATATGGGAATTTGGGTTGGACGGG
GTACGGATCGATTAGTCCATCCATCTAATGACTATAAAACACAATGCTAATATTTACA
GTGTTCAGTTACGGTACATTTGAGTTTTCTTACGCCTTGGGAATGGCTGTGATTTGCCT
GC**AGAAACATTCAGTGAACCTGATCCACATCGAGTCACGTTCGTCGCAGCGGTT**
CCCTAGCCAGTATGAGTTCATGGTGGAGTGTGCTCCAGGTGGAGACATCGGTGG
TGCTGTGACCAACTTACGGCAGAACTCCGCCTACTTCCAGATCATCTCGCGCAA
CCACAAGGACAACAGAGGTTAGTTGCCCACAGTAATGTAACGACAGAATCAGATAT
TAAATTATACCTTACGATTCAATCCTTTAATCTACCACCCTATACCTAATCTTTATGTA
CCTACCCAATACCTATTGTTCTGTCCTCCATTCTTTCCGTTTTTTTCACTGTCAATCTTT
TTATTGATGGCATCGAACTAAACTCCTATCCTTGCAAACCGACTATTTATTTCACTGA
AATGACCATTTATTTCACTTTTTATTTCATCGAACTGACTGTCTATCACTTCAAGCCGC
TTGTCCATAGTGGCGAGTTGCTGTAATACTACCATAATCAGATCTACTTTACCTTCAG
ACACTTCATCGTCAGTTGAATAGTTTTCTTTACTCTCTTCAAAATTCCCAGTTTTCATA
TCTACTGGTAGTTTATTCGTAATGTCATCGAAATTTGCCATAATTGCTGTTTCATCGTT
AACCTCAACACTTGTATCGAAAACTTTGTTACACGCCTGTCACGACGCAGTTTGTTGC
TAAAGAAAAATATAATATAGGTTACTGATCTTTTTTTCTTTGCTGTCTGTAGATTGTGG
TCTTTTTCACATCCAGTATATTGTCCCAGTCTATTTACATGCACAGTCAATTGATCATT
GTTTGTTCTTTCTCTGATTCGTAATACCACAGATAACACAGTTATTTATAAATACCTTG
CTTTTCAACTGAAAAAAAAATAATGCATTGTTCCAAACCCCCAGCTCAATTCAACCAG
TTGAAGTGTCCCAATATTTTGGTTGATTATTGGGAACACTACTATATAGTTCCATTGA
AAATTAATTTCTTATAAATCATTCTTAATTTATTTTTTTAAGTATCTTAGTGGAGTCAG
TAAATGAATGAAACAATATTTGTCTCAACTTCTTTTATTCAGATGTAGCTCCATCTAAC
TGACTAATATCAAGTAAAGTCAACATCAACTGAACCAACTTGAACTGAAAAGTAACT
GCACATCAGTATCTTAGTTCTATTTACACAATCTTGACAATATGATGTGTTAATACATT
GTTGTAATTGTTCAATTACAATGAAAGTTGATGGTTACAGTCCAGTTATTCGTTGATA
CACGTCTTTTGTATAATATGAACATTATTTCATCCTGAGTTACAAATGTTCATGTGACA
TACTCTAATTGTGACATTAATAATCATCTGGATATACTTTCAGAAATGTTCATCATCA

GCACAAAAATATACAGAGATGATTTTAAAGTTACATTTGTGATCTTTTCTACATACATTTTGGGTTTGAATTAATTTTTTATGATTTGCTTAATTGACAACTGGGACATTGACAACTGGGACATTGACAACTGTCTAGGGTTGACAAACCAAAACAAGACAGCAGAATTATATGATGGTTGTGAATATTGAGACAAAAGCAAACTGTATGTACAGTTTAGTCACTTGATGTACTTCCAAATGTCTACTTTATTTCATCATTGCAAAAATTGGCCCAATTATGTTTTCAATAAATGAAATGAGGCACTCGACCACCTGAAAAAATCTCAGAAGCAAATGACCTCTATATGATGCATTGCAAAGCTTAAAAGCATCCCTATATTAAAGCTGGTCCATTAGATTCTTATCAATTGCCCTAGTTAATATGAAGAGAAATGGCAAGATTGTAATTTTTCCCCTGATTCTTTATGCAAGTTGACCAGTTTCAACACTTAGGGGTGTCATCCTCTGGTCTTTGCTACAGTTGACATTTAACGGATTATTAAATCACTCCAAATATTATGCAGTTAAATGCCAATTGTAGCAGAGACAAGAAGATGACACCCTTAAGTGTCAAAACTGGTCATCTTGCATAAAGAATAAAAATTATGATCTTGGCATTTCTCTTTATATTAATATAATAAGATGGCTTCCTGTGATGAGTAAATAAAGTTAAACTTTCCTAGTGTTTAAATATAATGTTGTTAGCCTATACAAACTATAAGAAACAAGAGTTCAGAATGGATGACACAACTAGAAACACTTTGTCAGAATTAATAGTGGACTGTCACCTTATTCCTTTACTTCCACACATGCCCATTCAGAATCACCCAGCAGAAGTATAGCAGCGATTTATGGAATATTTCATATGATTTC**AGATACTGTTCCTTGGTTTCCACGCCGTATCCGTGACTTGGACAAATTTGCCAACCAGATCTTGTCTTATGGAGCTGAGTTGGATGCAGATCATCCTGGTTTCACTGACCCAGTGTACAGAGCAAGAAGAAAATATTTTGCTGATATTGCGTACAACTACAAACA**GTAAGTCACTTCAATCAATGAAGTATAGTCCTTAACCTACAACATTCATGCAACTGACATTATGAAGAAGGGAAAAAAATAGATGTTAAATATCTTTTTCTCTTCTAATACACGGAAACAAATCAGTATATATTGTACTTCAGGGATACTCAAGTTCTGTTGTAAAAACTGCCTCCAAACTGCAGGACTGTGACCTCAACGAAAGACTTAAAGAGCAATAGTCAGTTGCCAATGCCAAACAAATCTACCCTGTTAGAGCTAAAATGCTTTGTGAATCTCAAATTGGCTCATTTATTCTGTTGAAGCTATTGAAGCACATCTTCTGTGCTCTGAATATCTGCAAAACTGCAGCTTTATTGACAACTGAGAAAATTCCATTTTATTTCTAATCATACCTATACATATTGTTTGTAATTCATACTAATACATAGTTTTATTTCAGTCATGTTGACATTTTTCACACTTGTTCACTATGGGGGTCAGAACCACCTATGATACATAGTTCAGGAGACATGAAATTATAAGCAACGAAAT

GTCTGAAAATTGCTAGAACATATGATGTGTCAATAACAGTGATACTTTATTCATCTGC
TATTTGACAATGACAGCACTTAGTGACTTGCATATAATTTCAAATCTGAAATAAAAAT
TTTCTCCCTGATGACCTCAACAAAATGATGAAAGGAAAGAAGTTTATTGCTTATTACA
TTTTCACATTTCATCAAGTACAAGTACAGAATTTGACATGAATTTATTGCCTCTATACT
ACTAACTGTGTTCATGATACATATTACAGACAGTATGCACATATACCACTCAGGGTAC
ATGCAGAATTATATCATATAGTATGATACATAGCTTAGGAAGTGTGATGTTATTCACT
GTACCTTTAAGGTTCATGTAACTATCCAACCATGTATAATGTTTTAATTTATTAAATTG
TTACTACTTTTGTGGGTAGTATGAAGCTTCAAAATTAAATGATAGTATTACACATAGT
TGAGGAGAAATGACATAATAAACAATGAGATGCATGAAAAATTGCCACATCATATAT
CAGGTTTAATTTATAAATTTTTTACTACCAACTCTATTTGCAACGTATTTTGCAATTTC
CTAAGATGTAACTCTGTATGCCAGAGAAATTACATAATTTTAGGCCCCATTGTTTGGG
AGAGTTAACATTATAAGGCTTGAGATGCATGAAAATGAAACTGCAGTTCAGAATTTG
CTACAGATACAGGTGAAACCGAGAAAGTTATGGACAGATGTACCTAGGCAATATTTT
AAAGCTAGCCCTTGATACATGTAATAAGTTCATTTTAGTCTACAATTTCAGTAACTGT
GTAGCATCACTGCTTTCATACACTCCATAAGTTTGAGTCCATTAATATATGTATTTGTG
TTCATTTGACTCTTCAAATGCCATTTGACTGACAATATGAGGTAAAGGTTAAAGCAGC
TCCTCACACTTGAAATATTACATCAGTGCCATGCAAAGTGGATTGCTTAATACATCAC
TGACAACATTAACATCAAATTTTCCTTTTATTACCAGTGCTCATTAGGTACTATAGCA
ATAGATCCACATCGCAAAAATAACTGACATGTAAGAAACCTTTTTTCATAATCCAAAT
GATTCAAAAAAATATTGACACTCTTGGGATGGTAGCCAATTTCTAGTTGTAATGTATG
CACCAAAAGTTTATTTACATTCTTGTCAGCAAAGAACTTCAAAATTTTGTCAACCATA
ACAAACTAACTACTGTAATAAACTTTTATCACTCACCTACTAAGGATCAACTTATTAT
CCATTTTAAGCTGTTTATGTATTTTAATATGGCTACTTGTTTAACATAAAGTTGTATAA
GTAATGACAAATGGCTGCTTGCAGAATAAGGGCTAAGGCAGAATCCATTTTTCCCAT
ACCTCTAGCAGTGATTTGTGATATTCAAAATACCAAGAATCACCATGACACCAATTTC
ACATTTACCATGTTTCTTCATGACTGAACAGAGGAATGAAAATGGCTTGTGAGGGGC
GATCAAATGTCATATGATATTTTCCTGTTATTTAAAATCACATGTATTTTTGCATGAAA
CTTCCTTATAGCTGTTACTGAAGTGTTATTTGTATGCTCTTCCTTCCAG**TGGTGAACC**

TCTTCCTCATGTGGACTACACCAAGGAAGAGACTGAGACATGGGGAAAGGTGTT
CCGCGAGTTGACCAAGCTCTACCCAACGCATGCATGTCGTGAGCACAACCATGT
CTTCCCACTTCTCATAGAAAACTGTGGTTATCGTGAGGATAACATTCCACAACTT
CAAGATGTCTCAAACTTCCTTAAAGGTAAGCAGCACATTTTGTACATTATTTTTA
AAGCTCTGAATAACTTCATCAAAATCTACTGAAGGTTTCACACTCAATACCTAAG
TTCCAAATACATATGGAAAGATGTCACTAAACATCCACTCTGGCCCATTCATTCA
GACTCATGTCTAAATTTATTCACATTACCTAAAATTACTTATCTTCAGTTAATTTA
TTGGTGATGTATTTTCAAGTGGCTAGTCCACATATCTGTGTATCAAGGTTGTAAA
TTATTAGTTACAGTACCAGGAACTACCAGATTATCAGTAGTTCATTGAATAGGAG
TCCCACAAGAATTGTTGTCACCATATGCAAGCCTAATCCTGTGATAATGTTAGCA
TAGTGGGACTATAATGTTCATCTCAATATGATGTGTTGCGTTTGTTGGCAGACTG
CACGGGTTTCACACTGAGACCAGTAGCTGGTCTGCTGTCATCACGGGACTTCCT
GGCAGGGTTGGCTTTCCGTGTCTTCCATTCAACACAATATATCCGACATGCCAG
CTGCCCACTATACACCCCAGAACCTGATGTCTGCCATGAGCTGCTAGGCCATGC
ACCACTCTTTGCTGACCCTGCATTTGCACAGTTCTCACAGGAGATTGGTCTAGCC
TCCCTTGGTGCACCAGATGATTACATCGAGAAGCTTGCAACAGTGAGTATTGTA
GCTATCTTATAGGCTCCAAGTAGTATTGCACATTTGCTGACTAGGCTTTTAATGA
AAAAGTCACCCACTAGAAGTAAAGTTACTATAGTCTCTATATTCTATAAGTCCTT
CAGTATTGTCATTATCAAACAACTATACAAAAAGATTTAAAAAAAATACATAGCC
ATGTATACAATATTTAAAAAGAGTACATTTGTGCTCACTGTAAAGACAACACTCT
GAGCAGCATAGAGGCACAATAAGATCATCCTAACGAATGTTTGGCCAGTCTTAA
TGAGAAAAACAAGATAAACAAACACATAAATTCATACAGTCATATACATACAACT
CATGTGCATGCAAATGCATTGCATGCACGCCTTTTCTAATGAAGGCCTGGCTGA
AAGCTCATTTGAACTTTTTGCCTGCCTGCTTCTTGTCATGTCATCTTAACGGTGA
GTAACAATACATAATTTCCACAGTATTGTAGATGTACCAACCTGGACTTATATAA
TTGTTTGATTTAACCATAAGTAATGCAATGAAATTAGGTTACAAAGGACTACCTG
TCATTTGCAACATCCAGGTTTCTAATAGATAAACATAAATGGGAGTCAAATGAAA
ACACAGTGACTGCTTTGCACCTAGACATACATCTCAATATTCTTGAGGAAATTGT

AAACATTATACTGACAATTCATACAATACTCTAATTGACAGATTGGTTCAACCAT
AAGTGACATACTGGATATGTGGTTTCACACCTCTCTCATGGGACACTTGACAACT
ACATGAAAAAGCAAACTTTATAAGTCACATAATGTACACTGCCAAAACTTCTGCG
ATTTTTTCACAAATATATTTTATATAAACTAATTGTTTTGAAATGCAATATTGTAA
GACAAGGCCAGTAATATATAAGAGAGAGAGAAAAGATCGCAAGACAACCTGTCC
AAAAAACTAAGGTTATACTAGTATCTATTTCACATCATTTCTAATTTACCTTTTGT
TAGAACCTATCTACTCTATACATTGTTATTTTTCCACAATATTTTTTATTTTCATA
AAAAATTCAGACACTACCAATACTATTTTTACATTTTATGTCATATAATAGAGATT
ATCATAATATTTCTGTGCATAGCAATTTTAACCCCAAAGGTCACGTTGTGTGGTG
TAATGTGAGATGAAGATACACTATTATCCACCTTGATTGTGTGATCAACAAATCT
GTATATTCTCATAAAAAATAACAATGAACTCCTTATATCTGTATTATCACTCTGTA
TTAAATATTTAACTTTCTGAAGTAGGGAGCATATGACCTTTTTGTTAGTGAAATA
TACTGCTGCAAGTGATAACTTACAAACAGCTAAAGAACTACAGTTCAGATTTTAT
ATCATACATGCTAATGAATGCATATATGTATCTATACTGTTTCTATCAATGGAAG
GTTTATATTGGAATTCAATAATATTATGAGAATAGGGTGCAAATTGCCCCACAGA
TGTCAATGAACCACAGAAAAGCATGTAGAAAAGACAGTCACATTTGTACAATTTA
ACTTTTAGACCCAGATTTCATAAGAAATGAATGCTTACACATTCATAAAATCACA
GTGGCACTACTCACATACATGACTACCATCTCCAGTATATATATTGTGAGTCAGG
ATAGTGGCCAGAGAAGGCAGACATGTATGGATGAGTTGTTCCTGTATGATTGGT
GGAGGTTTATAGGATCGGGTCGGATAAGACACGAACACTCCAACTTTCATACAC
TGACTTTATTATAACACGTTTACACTATACATGTCAGGCGGGAACTGGTGTTTCC
GCTTCCTTACAGCAAAGACCTTTCGTAACACAGAAAGATACTACACTCGTCCAAA
GTTGTTAGGTATCGATTCCCACATAGCCCCCCCCGGCAGTATGGAGTACATCTAT
TCATGTGACGTATGTTCTTTCCTGATGAAGGCTATGCCCAAAAAATAAGTTTTGTGAA
TGTGACTGTCTTTTCTATATGCCTATTTGTAGCTTAGAAATGGTCTCTGTGGTGAGCTG
CAACCTTTCCCCATTATACTTTCTGTATAGTATGATATCACCATCTTGTGTTGCAATAA
GTGTTACAGTACATCTACACTGCAAGCCACCCACCATGTGATGTGTGGTGAGGGTTTA
TCATACCATTATTCCAACATGGTATATGAGGGGGAAAGGGGGGATGAGACAGTAAAG

TACATTTAATACACTACATATAAATGTTAAATGTCTAAATAATATTCCTTCATTTTTTG
TTTTAG**TGTTTCTGGTTCACTGTTGAGTTTGGACTATGCCGTCAAGACGGCCACT**
TAAAAGCTTATGGTGCTGGTTTGCTATCTTCCTTTGGAGAGCTGCAGTACAGCCT
GAGTGGAAAACCAGAACTAAGACCATTTGAACCATCAAAGACTGCAGAACAGAA
ATATCCTATAACAGAGTATCAACCAGTTTACTTTGTTGCAGAAAGTTTTGAAGAT
GCAAAAGAAAAAATGATGTGAGTAACAAATAACAATCATCGTTAATTTTATAATCAC
TGGCCAGTAATTGCTTTCAGAATGTGGAACTTTTAGTAGGTTTCATAGAAACACATAA
AAAGATGTTGTGTTTATGGTGTTCCCAAAAAATTAAATCCTTCATTGATTCATGGGTG
TTGCATTGAATTGAGTTGTGAGAACAACCTAGCCTCATTTCTCCACTTTTCTACTTAAC
CACAAAATACATTAGCAAGTCACAACAGCACCCAAAGATGAATATCAGCTATTTTAT
TGAAGTGTCAGGCTGTTTTCACAACACAATTCAATGTAGTACCTTTGATGCACTGACT
CACATTAAAGTATGTTACATGTTTCTGCTAGAAATAACGAGCTACATTTTAATGTGAC
TACTAACCACACTCACTTGTCTCTTCACGAACTTAACAAGGGTTTTTTTCTCTCTCTC
TCATTTCATATGTGTAAGTGTGTGTGTGTGTGTGTGTGAGAGAGAGAGAGAGAGAGA
GAGAGAGAGATGGAGGGAGGGGAAAGAGAGAGTAATTTAAAATTATGAAACAGAAT
TGCTGACCTCCACTTATCTTCAAGGCACAAATGTTCAATACTTATGATAAACACTTGA
TATGGTGTCTGTTCTTTGGGACATGTCTAAAAGAACAGACACCAAATCAGTCATTGCA
CATATGCACTAAAAGGAAGTTGACAATTTCTGTGAAGATGCACAGATATGCTTGAGT
CTTTGTGGGACTCCAGTAGAGGCTGTGAGCAAGAAGAAACTAATGGACAGGGGGCAC
TATGATCATAGTGTGCTATAAGTTGAGAATTTGGGCAGAAAGGGAAGCACACCTGGA
TAGCCTAATAGGTTAAGATGAATACTGGAGTGAAGTGGAAAATTCAGGTTCAAATGG
AAGTCCAGCACCAATTTTCAGTTTTGCTGTTGAATTTATTTAAGTGCGCTTAAACAGTT
GAAATGTTTCAATATTCCAAATTAACTAATCAGAGTACATCTATAGAAGATAAATAAC
TGCCAGTCAGCAATTCTGAGTAATACATTTATACTGTTCCAGAGAAAATATGATTTAA
TGTTTTCTTGTAGTCTGTGTGTAACCATTCAATTTTTTTTTCCACAG**CAAATATGCTCA**
CACAATTCCACGACCTTTTGGAGTGCGCTACAATCCATACACACAAAGTATTGAA
ATACTGGATTCAAAGCCTCAGATACAAACCTTGGTAGACAATATTAATGAAGAAA
TGCAAATTTTAATGGATGCCTTGCGAAAATTGTAAATCAGCAAACATAGTTCCTG

ATTTGTACCATACCAAAGCTGTAATATGTAGATAGATGTGTTAATATAATATTTCAAAGATCATAGATCAAACAATAA
