# Supplementary figures and images for: Long noncoding RNA PAHAL modulates locust behavioural plasticity through the feedback regulation of dopamine biosynthesis
Source: PLoS Genet. 2020 Apr 29;16(4):e1008771. doi: 10.1371/journal.pgen.1008771 (PMC7241820; doi:10.1371/journal.pgen.1008771)

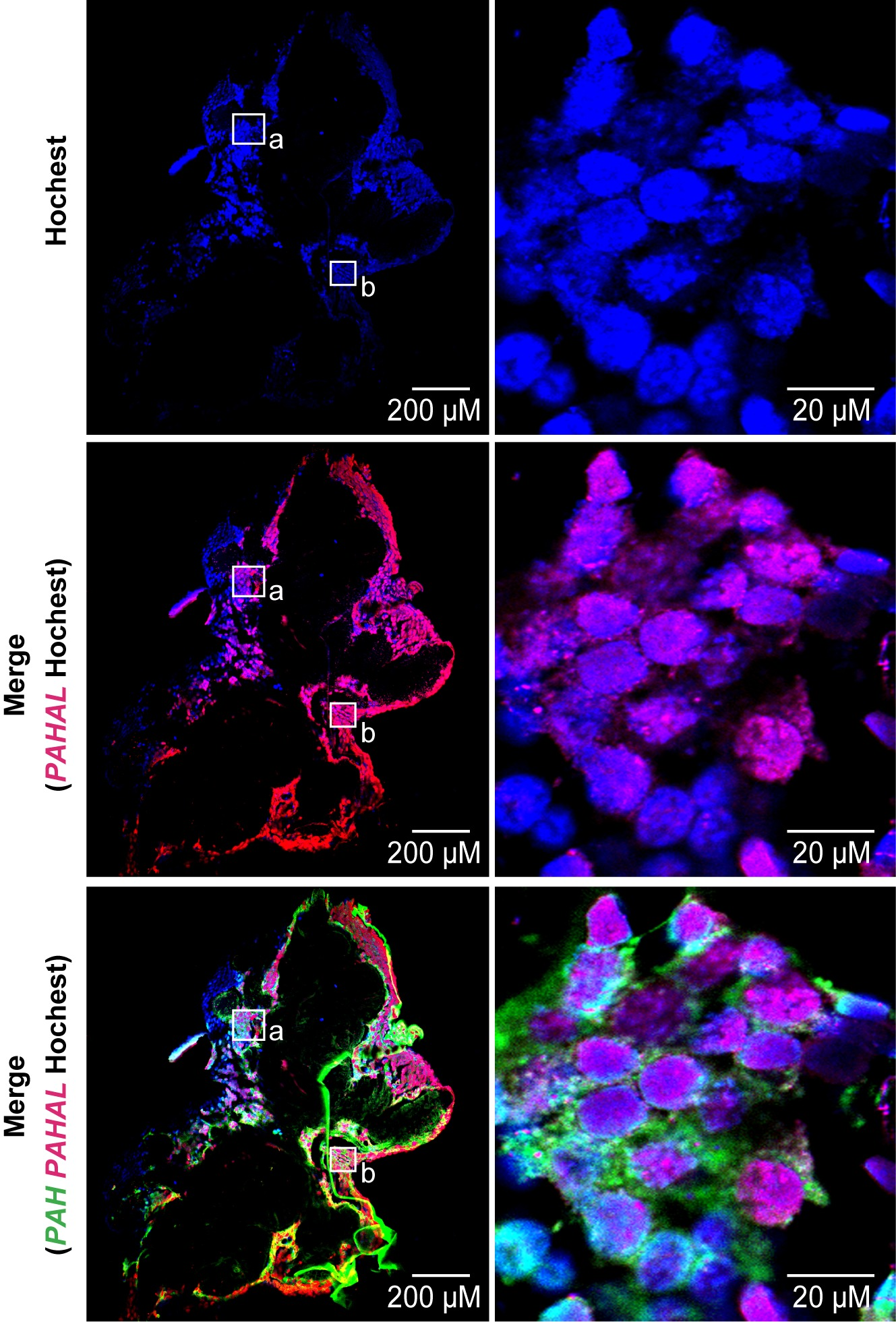

Supplement: S2 Fig — The nuclei of cell bodies in locust brain neurons are stained by Hochest 33342 (blue signal). A purple signal indicates that the nuclear location of PAHAL. 10× (left images) and 63× (right images) magnification. (TIF) [file pgen.1008771.s006.tif]

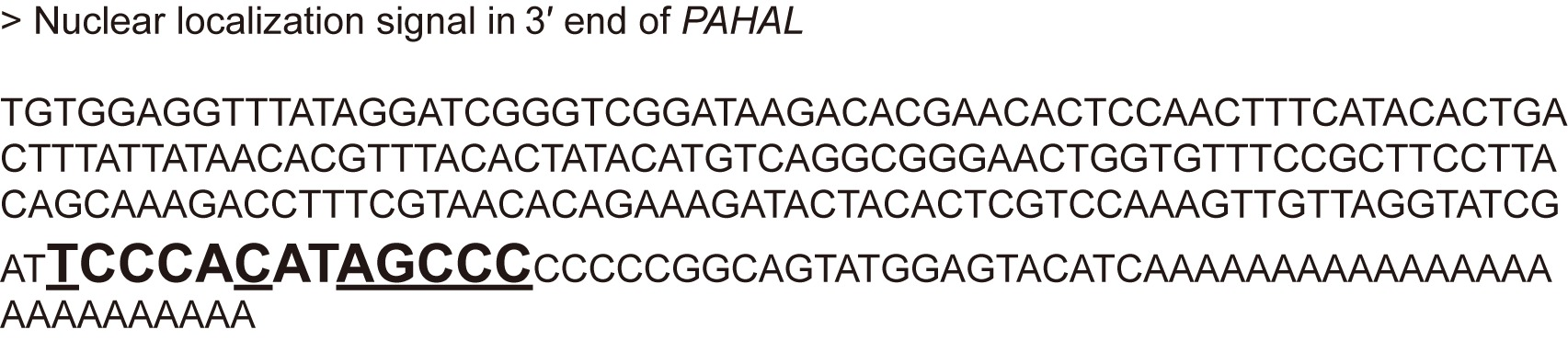

Supplement: S3 Fig — The bold text represents the NLS sequence “WNNNNSNNAGCCC” (W = A/T, S = G/C). The underscored characters represent the conserved nucleic acid sequence. (TIF) [file pgen.1008771.s007.tif]

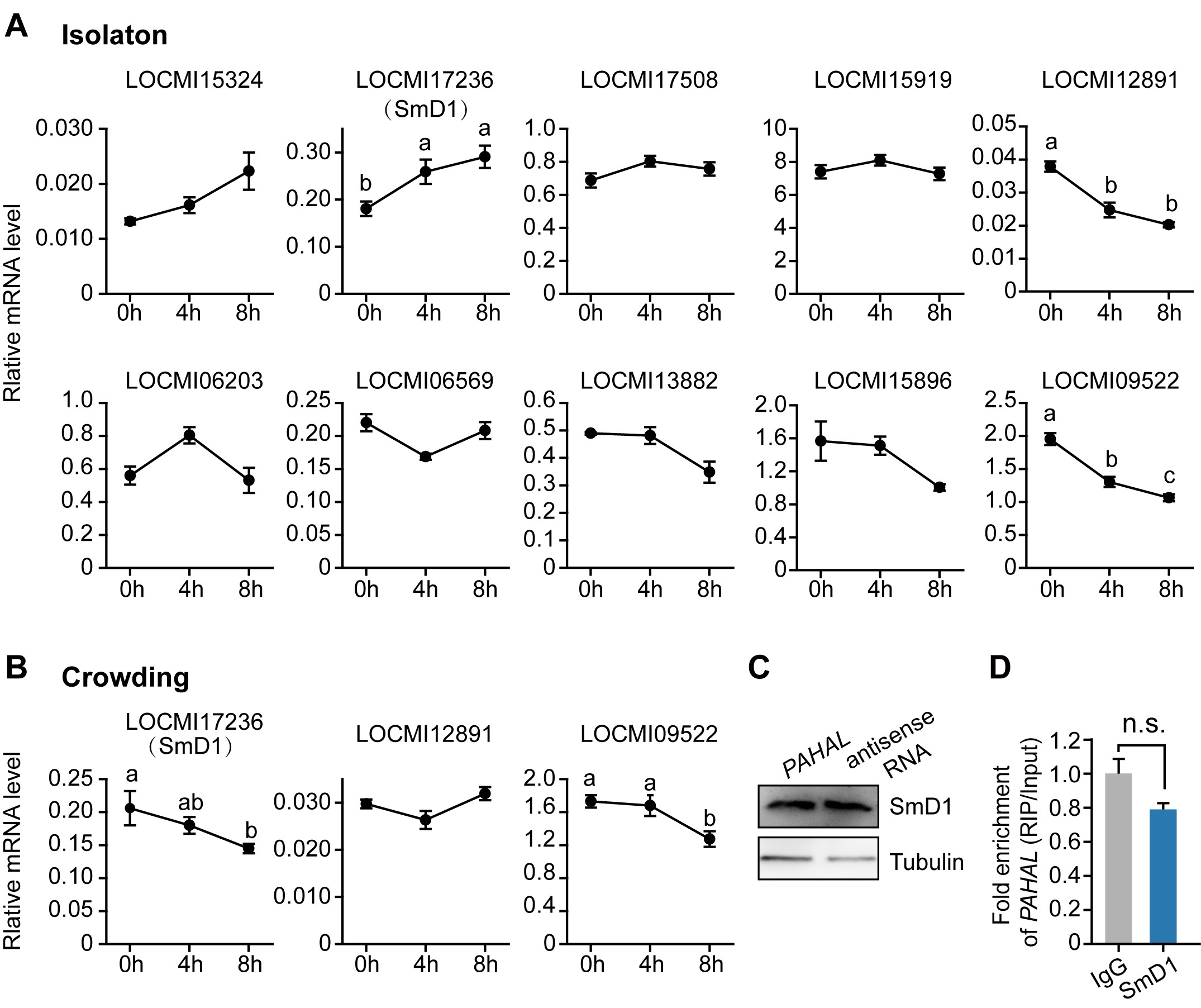

Supplement: S4 Fig — (A) mRNA levels of 10 proteins in nymphal brains during isolation. The 10 proteins (excluding SRSF2) identified from PAHAL pulldown pools. Fig 6C shows SRSF2. The mRNA level was determined through qPCR. The crowding responses of three candidate genes that showed changes in the expression in response to locust isolation were measured. (B) mRNA levels of the three candidate genes during nymphal crowding. The SmD1 gene (LOCMI17236) with prominent density response was chosen for further tests. Eight biological replicates of eight brains were prepared for each treatment. Means labelled with the same letter within each treatment are insignificantly different. (C) Western blot analysis for the detection of the nonspecific association of SmD1 with PAHAL. Tubulin was used as a control (n = 4). Four biological replicates of eight brains were measured in each treatment. (D) RIP in vivo revealed no association between SmD1 and PAHAL. Nymphal brains were harvested for RIP with SmD1 antibody or control IgG. Five biological replicates of 50 brains were prepared for each treatment. Error bars represent ± SEM. (TIF) [file pgen.1008771.s008.tif]

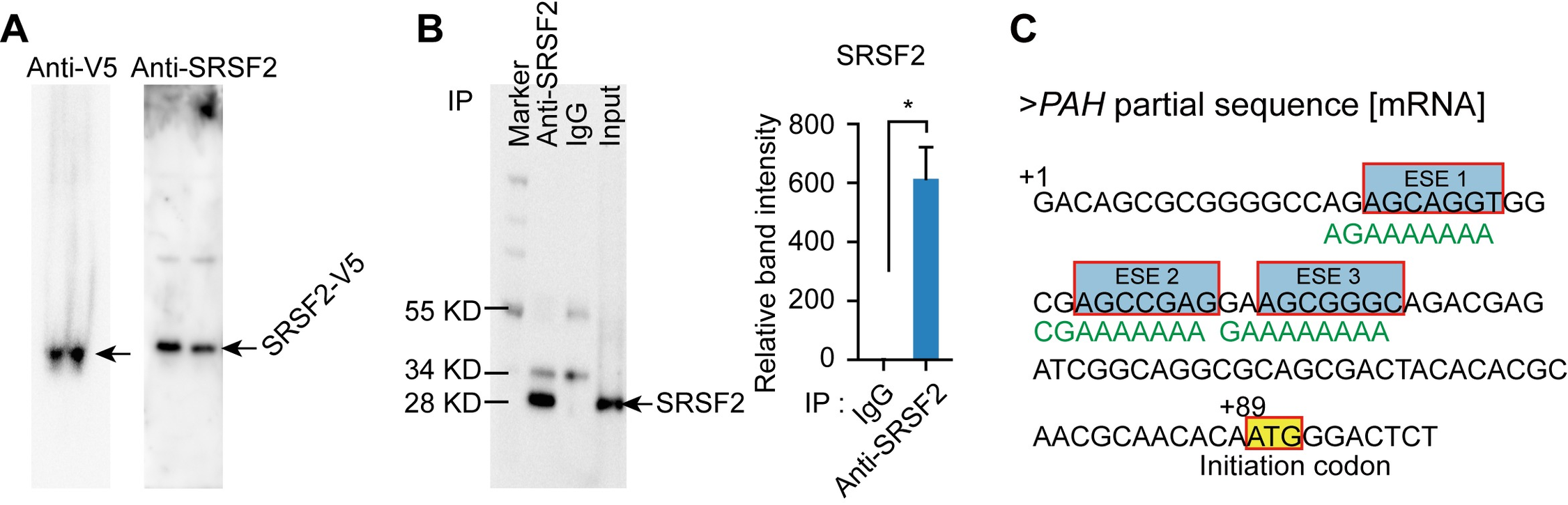

Supplement: S5 Fig — (A) Western blot analysis of locust SRSF2-V5 fusion protein. The protein was expressed in HEK 293T by transfecting with pcDNA3.1/V5-His/SRSF2 and detected using V5 antibody or the locust SRSF2-specific antibody. (B) SRSF2 was detected in lysed brains immunoprecipitated with the locust SRSF2-specific antibody or IgG control. Data were normalised to IgG. Five biological replicates of 50 brains were measured. Student’s t-test: *P < 0.05. Error bars indicate ± SEM. (C) Distribution of SRSF2 binding elements (ESEs) in the promoter-proximal region of PAH. The three predicted ESEs, namely, ESE1, ESE2 and ESE3, in the 5′-UTR of PAH gene are highlighted. “+1” represents the TSS of PAH. “+89” represents the end of 5′-UTR of PAH. The green characters represent the mutant sequence of ESE. Student’s t-test: *P < 0.05, **P < 0.01; n.s., insignificant. (TIF) [file pgen.1008771.s009.tif]
