# Supplementary material for: Long noncoding RNA PAHAL modulates locust behavioural plasticity through the feedback regulation of dopamine biosynthesis
Source: PLoS Genet. 2020 Apr 29;16(4):e1008771. doi: 10.1371/journal.pgen.1008771 (PMC7241820; doi:10.1371/journal.pgen.1008771)
Supplement: Attachment — Submitted filename: Response_to_Reviewers-zx321.docx [file pgen.1008771.s010.docx]

Dear Drs. Ewer and Copenhaver,

Thank you for your organizing the review for our manuscript entitled 'Long noncoding RNA *PAHAL* modulates locust behavioural plasticity through the feedback regulation of dopamine biosynthesis' (PGENETICS-D-20-00072). I thank all reviewers for their constructive comments, which are valuable for improving the quality of our manuscript. The point-to-point responses to each reviewer’s comments are attached. Numerical data that underlies graphs and sample image data have been uploaded to Harvard Dataverse Network with the websit <https://dataverse.harvard.edu/dataset.xhtml?persistentId=doi:10.7910/DVN/LXRBKJ> and <https://dataverse.harvard.edu/dataset.xhtml?persistentId=doi:10.7910/DVN/KSVYWY>.

In addition, we modified one of the affiliations of the author Xia Zhang. Page references in our reply below refer to the revised manuscript.

Please do not hesitate to let me know if you have any question about this paper. Thank you very much again for your attention to our paper．

Sincerely yours,

Le Kang, Dr., Professor,

Institute of Zoology, Chinese Academy of Sciences

Beichen West-road 1,

Beijing 100101

P.R. China

Tel: 86-10-64807399

E-mail: [lkang@ioz.ac.cn](mailto:lkang@ioz.ac.cn)

Decision on your article submitted to PLOS Genetics (PGENETICS-D-20-00072) - [EMID:edb45ed4c1df3521]

* Please note while forming your response, if your article is accepted, you may have the opportunity to make the peer review history publicly available. The record will include editor decision letters (with reviews) and your responses to reviewer comments. If eligible, we will contact you to opt in or out. *

Dear Dr Kang,

Thank you very much for submitting your Research Article entitled 'Long noncoding RNA *PAHAL* modulates locust behavioural plasticity through the feedback regulation of dopamine biosynthesis' to PLOS Genetics. Your manuscript was fully evaluated at the editorial level and by independent peer reviewers. The reviewers appreciated the attention to an important topic but identified some aspects of the manuscript that should be improved, and which may require a re-review of the revised manuscript.

We therefore ask you to modify the manuscript according to the review recommendations before we can again consider your manuscript for acceptance. Your revisions should address the specific points made by each reviewer.

In addition we ask that you:
1) Provide a detailed list of your responses to the review comments and a description of the changes you have made in the manuscript.
2) Upload a Striking Image with a corresponding caption to accompany your manuscript if one is available (either a new image or an existing one from within your manuscript). If this image is judged to be suitable, it may be featured on our website. Images should ideally be high resolution, eye-catching, single panel square images. For examples, please browse our [archive](http://www.plosgenetics.org/article/browse/volume). If your image is from someone other than yourself, please ensure that the artist has read and agreed to the terms and conditions of the Creative Commons Attribution License. Note: we cannot publish copyrighted images.

We hope to receive your revised manuscript within the next 30 days. If you anticipate any delay in its return, we would ask you to let us know the expected resubmission date by email to [plosgenetics@plos.org](mailto:plosgenetics@plos.org).

If present, accompanying reviewer attachments should be included with this email; please notify the journal office if any appear to be missing. They will also be available for download from the link below. You can use this link to log into the system when you are ready to submit a revised version, having first consulted our [Submission Checklist](http://journals.plos.org/plosgenetics/s/submit-now#loc-submission-checklist).

While revising your submission, please upload your figure files to the [Preflight Analysis and Conversion Engine](http://pace.apexcovantage.com/) (PACE) digital diagnostic tool. PACE helps ensure that figures meet PLOS requirements. To use PACE, you must first register as a user. Then, login and navigate to the UPLOAD tab, where you will find detailed instructions on how to use the tool. If you encounter any issues or have any questions when using PACE, please email us at [figures@plos.org](mailto:figures@plos.org).

Please be aware that our [data availability policy](http://journals.plos.org/plosgenetics/s/data-availability) requires that all numerical data underlying graphs or summary statistics are included with the submission, and you will need to provide this upon resubmission if not already present. In addition, we do not permit the inclusion of phrases such as "data not shown" or "unpublished results" in manuscripts. All points should be backed up by data provided with the submission.

PLOS has incorporated [Similarity Check](http://www.crossref.org/crosscheck.html), powered by iThenticate, into its journal-wide submission system in order to screen submitted content for originality before publication. Each PLOS journal undertakes screening on a proportion of submitted articles. You will be contacted if needed following the screening process.

To resubmit, you will need to go to the link below and 'Revise Submission' in the 'Submissions Needing Revision' folder.

Please let us know if you have any questions while making these revisions.

Yours sincerely,

John Ewer
Associate Editor
PLOS Genetics

Gregory P. Copenhaver
Editor-in-Chief
PLOS Genetics

--------------------
Reviewer's Responses to Questions

Comments to the Authors:
Please note here if the review is uploaded as an attachment.

# Reviewer #1: Summary

Zhang et al. have presented an interesting and thorough mechanistic investigation into the role of phenylalanine hydroxylase (PAH) in brain biogenic amine metabolism and transcriptional regulatory networks. The aggregation switching behavior in locusts has been studied from a variety of perspectives and the authors build upon a significant number of studies that specifically indicate the role of dopamine and biogenic amines in this phenotypic transition. I found this paper in general to be methodologically strong and integrative, and the experiments across domains (targeted / high-throughput gene expression analysis, metabolic measurements, PCR, behavioral analysis) were linked clearly. The transcriptional activation experiments were very nice, and demonstrated that the authors were committed to exploring the exact functional of the *PAHAL* lncRNA – they passed a high level of evidence to show that the *PAHAL* transcriptional activation was relevant in multiple contexts (rather than e.g. simply identifying a putative lncRNA transcript from RNA-seq data and publishing a hypothesis paper). My comments are all in service of clarifying the experiments, results, and interpretations of the authors.

## Main comments:

I am somewhat confused by Figure 2B. Usually, lncRNA regulate loci in *trans*, and are transcribed from loci that are distal to lncRNA targets (see comment 2 though). However Figure 2B looks like *PAHAL* has a transcriptional start site within the 4th intron of *PAH*, is transcribed in the same direction as *PAH* (e.g. at Line 102: “ The transcription direction of this gene [*PAHAL*] is the same as that of the canonical *PAH* (Fig 1B)”), and includes the 4th intron + 5th exon + part of the 5th intron (the red section). Is this the case? If so, then *PAHAL*’s sequence would be anti-sense to *PAH* (e.g. the *PAHAL* lncRNA would anneal to the sense strand of *PAH* so it would have sequence homology to the anti-sense *PAH*). Then I am confused by the primer design in Figure 1C -- the common reverse primer #11 is located within *PAH* exon 6, which is not within *PAHAL* as visualized, but apparently this means that Exon 6 is within *PAH* (as discussed in text, e.g. At Line 301). At Line 101 it is written: “The *PAHAL* sequence covers the sixth exon and part of the two introns flanking the exon in the *PAH* gene (Fig 1B and S1 Fig).”. First off, this not what I see in Figure 1B (Line 923: “The red shading indicates the location of *PAHAL* in *PAH* locus)”, where the red *PAHAL* transcript appears to be from the 4th intron to the middle of the 5th intron (and thus doesn’t include the 6th exon as suggested at Line 101). Then the Supplemental Figure S1 appears to be a screenshot of a fasta file of the sequence of the 3' end of *PAHAL* transcript, highlighting the NLS sequence, but not providing any information about which introns/exons of *PAH* are included within *PAHAL* (this Figure S1 should be more clearly informative, as it is used to support the idea at Line 101 “The *PAHAL* sequence covers the sixth exon and part of the two introns flanking the exon in the 102 *PAH* gene (Fig 1B and S1 Fig)”, which I am not seeing). So honestly I cannot tell exactly where the *PAHAL* locus/transcript begins and ends. For example in Figure 1C, the PCR with 10+9 gave no product, when this is what spans Exon 5 to Intron 5, I’d expect this to form a product with the unspliced *PAH* or *PAHAL* transcript. The caption mentions that the locus diagrams are not to scale – I think for a complex focal loci in this paper, a scale figure would be more appropriate.

Response:

We thank the reviewer for his or her careful reading and critical comments. We are sorry for the confusing illustration of the structure of *PAH* and *PAHAL*. In fact, the *PAH* locus harbors an 89-bp 5’UTR that is neighbored by the 47-bp exon 1. We now drew the two parts proportionally in the gene structure. We also added labels to all the eight exons (i.e., E1-8) in the Fig. 1B to avoid the confusion. You can see that the *PAHAL* sequence covers the exon 6 and part of the intron 5 and 6 flanking the exon in the *PAH* gene.

From the new structure illustration in Fig. 1B and the sequence information provided in the new Fig. S1, you will find that *PAHAL* has a transcriptional start site within the fifth intron of *PAH*. *PAHAL* is transcribed in the same direction as *PAH*, which were verified through using 5’- and 3’-end RACE to obtain full-length transcript (Fig. 1B and S1), using 5′ exonuclease digestion assay to detect 5′-triphosphate cap of *PAHAL* (Fig 1D) and using RT-PCR to identify Poly(A) tail of *PAHAL* (Fig 1E). Thus, we concluded that *PAHAL*’s sequence is sense to *PAH*. According to the reasoning, we re-organized the sentences in this paragraph (See lines 106 and 111). We have added a sentence in the figure legend to state “The bent arrows indicate the transcriptional orientation of *PAH* and *PAHAL*” (See line 945).

We’d like to accept the reviewer’s suggestion and provide the full sequence of the *PAH* locus that covers the sequence of *PAHAL* in Supplemental Fig. S1. In the figure, we highlighted the exon sequence of *PAH* and the full-length sequence of *PAHAL*. We hope that the present Fig. 1 and S1 can tell exactly the relative location of *PAH* vs *PAHAL* locus/transcript.

The PCR with the primer 9 and 10 gave no product because the primer 9 (has been re-numbered 11 in the revised version of the manuscript) is located within *PAHAL* but not in *PAH* transcript, and the primer 10 (has been numbered as primer 12 in the revised version of the manuscript) is located within *PAH* transcript but not in *PAHAL*. Note that we have re-numbered some primers in the revised version of the manuscript. The reviewer also mentioned the unspliced transcripts of *PAH* or *PAHAL* that may be used as PCR template. We don’t think a PCR product can be yielded from the unspliced transcripts because of at least two reasons: First, the unspliced transcripts are mostly rapidly processed into mature transcript and present at very low quantity in the sample; Second, the PCR product from the unspliced transcript is expected to be 2,320 bp, which cannot be amplified within 40 s according to the PCR protocol we used. We have added the method details (See lines 430 - 433 in *Methods*).

We are glad to follow the reviewer’s suggestion to draw proportionally to the gene length in Fig. 1B. We added a scale (in red) to the gene structure of *PAHAL* and had added a scale (in black) to the gene structure of the entire *PAH* locus.

Related to my comment 1: I appreciated the citations in Lines 295-299 about cis-regulatory lncRNA, but are there any citations to which % of lncRNA act in this manner? Otherwise these two cited examples could be the two rare exceptions. Or it could be discussed why we do not / cannot know which fraction of lncRNA may act in this way.

Response:

We don’t find reference reporting the specific percentage of lncRNAs that act in *cis*-regulatory manner. However, many studies have revealed the prevalence of lncRNA-mediated cis-regulation on nearby transcription (Yan et al., 2017; Gil and Ulisky, 2020). LncRNAs with *cis*-regulatory roles may arise from diverse ways, either from independent transcriptional units or overlapping protein-coding genes, for example, promoters or enhancers-associated lncRNAs, antisense transcripts or RNAs arising from within introns or near transcription start sites (TSSs) of other host genes (Gil and Ulisky, 2012). As an example, 50–70% of sense transcripts have antisense partners. And about half fraction of antisense transcripts are noncoding RNAs (Gil and Ulisky, 2012). In another study, the researchers used genetic manipulation in mouse cell lines to dissect 12 genomic loci that produce lncRNAs and found that 5 of these loci influence the expression of a neighboring gene in *cis* (Engreitz et al., 2016). These studies and many functional investigations on specific lncRNAs showed the prevalence of *cis*-regulatory lncRNAs. To address this point, we add sentences to the manuscript to read:

“These lncRNAs with *cis*-regulatory roles may arise from diverse ways, such as from promoters or enhancers, antisense transcripts or from within introns or near TSSs of other host genes [25]. For example, about half fraction of antisense transcripts are noncoding RNAs [25]. In another study, genetic manipulation in mouse cell lines revealed that, among 12 genomic loci that produce lncRNAs, five loci influence the expression of a neighboring gene in *cis* [24]. These studies have revealed the prevalence of lncRNA-mediated cis regulation [50,51].” (See lines 295-300)

Related references:

Yan P, Luo S, Lu JY, Shen X. *Cis*- and *trans*-acting lncRNAs in pluripotency and reprogramming. Curr Opin Genet Dev. 2017; 46: 170-8. https://doi.org/10.1016/j.gde.2017.07.009 PMID: 28843809

Gil N, Ulitsky I. Regulation of gene expression by *cis*-acting long non-coding RNAs. Nat Rev Genet. 2020; 21: 102-17. <https://doi.org/10.1038/s41576-019-0184-5> PMID: 31729473

Guil S, Esteller M. *Cis*-acting noncoding RNAs: friends and foes. Nat Struct Mol Biol. 2012; 19: 1068-75. <https://doi.org/10.1038/nsmb.2428> PMID: 23132386

Engreitz JM, Haines JE, Perez EM, Munson G, Chen J, Kane M, et al. Local regulation of gene expression by lncRNA promoters, transcription and splicing. Nature. 2016; 539: 452-5. <https://doi.org/10.1038/nature20149> PMID: 27783602

This is a comment about the RNAi experiments and interpretation. Line 116: “We knocked down *PAHAL* through dsRNA and siRNA interferences to test the regulatory relationship between *PAHAL* and *PAH* in the locust brain.”. I think that the unique relationship of *PAH*-*PAHAL* makes the dsRNA/siRNA experiments inconclusive, or at least not completely explained. Here is my contention: since dsRNA targeting the *PAHAL* locus also (by definition) targets the *PAH* locus, the dsRNA is directly knocking down *PAH* mRNA (as well as *PAHAL*) – in fact both transcripts are reduced an almost-identical amount, and follow the same regulatory time course in later experiments. The authors interpret their knockdown experiment solely in terms of “the dsRNA knocks down *PAHAL* mRNA, and the reduction of *PAHAL* mRNA reduces *PAH*”. However it is more parsimonious to suggest that the dsRNA corresponding to the *PAH*/*PAHAL* locus knocks down transcription of that entire locus, thus not necessarily acting via the transcriptional loop hypothesized. The piece I might be missing here is if the siRNA causes a “*PAHAL*-specific knockdown”, as suggested at Line 120. But again, any siRNA corresponding to *PAHAL* would correspond to an intron/exon of *PAH* and thus be likely to knock down *PAH* transcription directly – in other words, the entire logic of this section/experiment is that the dsRNA/siRNA experiments only knock down *PAHAL* (hence interpreting *PAH* decreases as mechanistically downstream of *PAHAL*), but because *PAHAL* is inside *PAH*, any RNAi-type experiments would be more simply interpreted as a transcriptional silencing of the *PAH* locus directly. I believe the correct control (or at least an experimental group to include in these experiments) for these dsRNA experiments would be a dsRNA/siRNA targeting a region in *PAH* but not *PAHAL* (e.g. in 1st exon of *PAH*). This would hypothetically reduce *PAH* without reducing *PAHAL*, and if this phenocopies the “*PAHAL* knockdown” presented here, would be strong support for the author’s interpretation. Also as a related note, just because a RNAi knockdown reduces a metabolite X, this does not mean that that the knocked down gene is a rate-limiting step in the synthesis of X.

Response:

The point that the reviewer raises is clearly an important one. We sympathize with the reviewer’s concern about the possibility of knocking down transcription of that entire locus by RNAi through dsRNA or RNAi corresponding to the *PAH*/*PAHAL* locus, even though *PAH*-specific or *PAHAL*-specific guiding RNAs were used. To address this question, we followed the reviewer’s suggestion and added control experiment (which we had done before the manuscript submission). Specifically, we performed dsRNA knockdown targeting the first three exons of *PAH* which do not cover *PAHAL* (using primer 7 and 8). Our data show that *PAH* knockdown significantly repressed *PAH* mRNA levels but had no effect on *PAHAL* levels (Fig 2C). We have added the new data to the text: “However, *PAH* knockdown had no effect on *PAHAL* level (Fig 2C)” (Lines 129 and Fig. 2C).

We accept the reviewer’s opinion that it is inaccurate to consider PAH as a rate-limiting enzyme in the synthesis of Dopamine, and give detailed reply in response to a related comment below.

I think that the sentence at Line 293-294, that the positive transcriptional influence of *PAHAL* was verified in “locust, fruit fly, and mice in vivo and in virto” is extremely strong. I think that this framing could be introduced earlier – that the effect of any candidate genes identified through high-throughput approaches would be verified through in vivo and in vitro experiments in multiple species.

Response:

Based on the reviewer’s suggestion, we put the sentence “the effect of any candidate genes identified through high-throughput approaches would be verified through *in vivo* and *in vitro* experiments in multiple species” in the first paragraph of the discussion (See line 276) and to make it clearer, we amended the discussion (See line 288 -313).

The SRSF2 experiments were very nice, and good support for the article’s main hypotheses.

A large body of research suggests that the rate limiting step of Dopamine biosynthesis is Tyrosine Hydroxylase (TH), and TH is used commonly as the defining marker of dopaminergic neurons (e.g. to provide just one citation: ncbi.nlm.nih.gov/pmc/articles/PMC3065393/ ). In the Figure 1a of this manuscript, it is clearly laid out that TH and DDC are both required after PAH for DA synthesis. At Line 72 the authors write that PAH “is a rate-limiting enzyme in the synthesis of DA and other bioamines [34,35]”, but citations 34 & 35 do not specifically support the idea that PAH is indeed rate-limiting for DA or any amine other than Tyrosine. In the Discussion from Lines 259-266 there are good citations relating to the specific role of PAH in locust, these citations could be relevant in the intro as well. Otherwise, I believe this statement that links PAH directly as rate-limiting to DA synthesis is inaccurate (it is TH, not PAH). In general the manuscript is oriented towards the specific role of PAH in DA synthesis, and this ends up neglecting non-PAH players in DA metabolism & PAH’s role in other processes.

Response:

We agree with the reviewer’s opinion that the rate limiting step of dopamine biosynthesis is tyrosine hydroxylase (TH) and not PAH in general. In fact, PAH is a dual function enzyme, that hydroxylates both phenylalanine to tyrosine and tryptophan to 5-hydrotryptophan (5-HTP), the precursor of serotonin. Therefore, PAH is involved in the synthesis of DA as well as other bioamines in brain and neurons. However, as the reviewer agreed with, PAH exhibits specific important role in DA biosynthesis and behavioral regulation in the locust. This specific role of PAH is validated by *PAHAL* inhibition in present study and expression manipulation of a miRNA targeting *PAH* gene in the locust (Yang et al., 2014). We thus made several revisions in the manuscript. We rephrased the sentence in the Introduction, and replaced one reference by another. Now the revised sentence reads: “Phenylalanine hydroxylase (PAH, also known as Henna in *Drosophila* and *Locusta*) catalyses the synthesis of tyrosine from phenylalanine and affects the production of DA and other bioamines in brain [34-37] (Fig 1A)” (Daubner et al., 2011; Coleman and Neckameyer, 2004; Yang et al., 2014; Ruiz-Vazquez and Silva, 1999). (Lines 72 - 74). We now emphasize the specific role of *PAH* in locusts in Introduction, and tune down the direct link between PAH and Dopamine biosynthesis. We have made revisions to improve the manuscript as follows: “These results indicate that *PAHAL* affects the DA synthesis in the catecholamine metabolic pathway.” (Line 149)“*PAH* expression affects the catecholamine metabolic pathway (e.g., DA synthesis) and controls the behavioural phase shift of locusts [38,39]. Specifically, miR-133 targeting *PAH* coding region repressed *PAH* expression, reduced DA production and drove locust soliterization while activated, and vice versa while inhibited [36].” (Lines 85 -87) “However, the transcriptome sequencing and LC–MS measurements illustrate that *PAHAL* predominantly affects the DA synthetic pathway in the locust brain (Fig 2), although the results do not exclude non-*PAH* players in DA metabolism and *PAH*’s role in other processes.” (Lines 282 - 285) “The *PAHAL* transcription then triggers the fine tuning of the *PAH* expression and in turn the downstream biosynthesis of specific neurotransmitters such as the DA that controls the aggregation behaviour of locusts (Fig 8).” (Lines 316 - 319)

Related references:

Yang M, Wei Y, Jiang F, Wang Y, Guo X, He J, et al. MicroRNA-133 inhibits behavioral aggregation by controlling dopamine synthesis in locusts. PLoS Genet. 2014; 10: e1004206. 10.1371/journal.pgen.1004206 PMID: 24586212

Daubner SC, Le T, Wang S. Tyrosine hydroxylase and regulation of dopamine synthesis. Arch Biochem Biophys. 2011; 508: 1-12. 10.1016/j.abb.2010.12.017 PMID: 21176768

Coleman CM, Neckameyer WS. Substrate regulation of serotonin and dopamine synthesis in Drosophila. Invert Neurosci. 2004; 5: 85-96. <https://doi.org/10.1007/s10158-004-0031-y> PMID: 15480914

Ruiz-Vazquez P, Silva FJ. Aberrant splicing of the *Drosophila melanogaster* phenylalanine hydroxylase pre-mRNA caused by the insertion of a *B104/roo* transposable element in the *Henna* locus. Insect Biochem Mol Biol. 1999; 29: 311-8. <https://doi.org/10.1016/S0965-1748(99)00002-8> PMID: 10333570

In general, whenever a P value is presented, I would appreciate full statistical information (e.g. Line 162 and 168 mention the statistical test used, add information on the N, and test statistic). In many other cases, I was unclear which statistical test was being performed, and how large the sample sizes were, without combining back through Methods/Materials/etc.

Response:

We gladly follow this suggestion, and have added the information on statistic test, the *P* value and the sample sizes in the revised manuscript (See lines 126 – 269, and other places appropriate).

## Minor comments:

Line 20: As is, this sentence sounds like *PAHAL* is what "is known for extreme behavioral plasticity", not the locusts. To clarify the sentence, something like "...behavioural adjustment in migratory locusts, a species with extreme behavioral plasticity". Also the latin species name could be included in the abstract, for search reasons and clarity.

Response:

Thanks for pointing it out. To avoid any misunderstanding, we revised the sentence as suggested to read: “Here, we report the regulatory mechanism of the nuclear-enriched lncRNA *PAHAL* for dopamine biosynthesis and behavioural adjustment in migratory locusts (*Locusta migratoria*), a species with extreme behavioral plasticity.”(Line 22)

Line 22, 42, elsewhere: Repeatedly this lncRNA is described as being “is sense to *PAH* gene”. This use of the word “sense” may be obscure for those who are unfamiliar with the sense/anti-sense terminology. Perhaps in an early mention, something like “*PAHAL* has sequence homology to the sense (coding) strand of the *PAH* locus” or “*PAHAL*” (or whatever accurate statement would clarify my uncertainty in the next sentences).

Response:

The point is well taken, and we modified the phrasing in the first mention in Abstract: “*PAHAL* is transcribed from the sense (coding) strand of the gene encoding phenylalanine hydroxylase (*PAH*), which is responsible for the synthesis of dopamine from phenylalanine. (Line 24) We also defined “sense” in the Author Summary:

“*PAHAL* that is transcribed from the coding strand of the *PAH* gene in the locust (i.e., sense lncRNA)” (Line 41).

Line 77: Would be helpful to add an estimate of how long "rapidly shift their behaviours" is referring to. E.g. does "rapidly" mean 1 hour, 1 day, 1 week.

Response:

We added the description as the reviewer suggested:

“Specifically, G nymphs exhibit significant behavioural solitarization upon 1 h isolation and full solitarization upon 16 h isolation. S nymphs display significant behavioural gregarization upon 32 h crowding [42].” (Lines 80 - 82)

Related references:

Guo W, Wang X, Ma Z, Xue L, Han J, Yu D, et al. *CSP* and *takeout* genes modulate the switch between attraction and repulsion during behavioral phase change in the migratory locust. PLoS Genet. 2011; 7: e1001291. <https://doi.org/10.1371/journal.pgen.1001291> PMID: 21304893

Line 128-131: This sentence makes it sound like the phenylethylamine (tyrosine/dopa/dopamine/octopamine) and tryptamine (5HTP/serotonin) biogenic amines are all downstream of PAH. Rather, as pointed out a few lines later, there are two separate branches (one downstream of phenyalanine/PAH, and one downstream of tryptophan). The authors then claim at Line 135 that “These results indicate that *PAHAL* only affects the DA synthesis in the catecholamine metabolic pathway”, which is incorrect – the knockdown of *PAH* (whether directly or via *PAHAL* transcriptional loop) resulted in changes to tyrosine plus some phenylethylamines (l-dopa/dopamine but not octopamine/tyramine), and also changes to 5HTP levels (why do the authors think that this tryptamine would be decreased by *PAH* knockdown when it is not linked to phenylethylamine metabolism?). So while it is true that “*PAHAL* only affects the DA synthesis in the catecholamine metabolic pathway”, this section would benefit from an explanation or interpretation of the unexpected changes to 5HTP on the non-catecholamine branch.

Response:

We appreciate the reviewer’s comment and would like to respectfully disagree with him or her on this count. Indeed, we agree with the reviewer that, in vertebrate, (TRH) and PAH are two distinct enzymes, with non-overlapping tissue distribution and involved in the two separate branches (Coleman and Neckameyer 2004). However, at least in *Drosophila*, *PAH* (also referred to as *DTPH* or *Henna*) encodes a dual function enzyme, that hydroxylates both phenylalanine for tyrosine synthesis and tryptophan for the synthesis of 5-hydrotryptophan (5-HTP), the precursor of serotonin ( Neckameyer and White, 1992; Coleman and Neckameyer, 2004). PAH exhibits different hydroxylase based solely on substrate, i.e., phenylalanine or tryptophan (Coleman and Neckameyer 2004). Regulatory control, such as end product inhibition and phenylalanine phosphorylation, is used for phenylalanine substrate, but not for tryptophan in *Drosophila* (Coleman and Neckameyer 2005). We therefore considered that the phenylalanine (tyrosine/dopa/dopamine/octopamine) and tryptamine (5HTP/serotonin) biogenic amines are the possible downstream of PAH in insects (Fig 1B). We added the related references to the sentence: “*PAH* encodes a dual function enzyme, that hydroxylates both phenylalanine for tyrosine synthesis and tryptophan for the synthesis of 5-hydrotryptophan (5-HTP), the precursor of serotonin in insects [34,44,45].” (Lines 139 - 141)

Related references:

Neckameyer WS, White K. A single locus encodes both phenylalanine hydroxylase and tryptophan hydroxylase activities in *Drosophila*. J Biol Chem. 1992; 267: 4199-206 PMID: 1371286

Coleman CM, Neckameyer WS. Substrate regulation of serotonin and dopamine synthesis in Drosophila. Invert Neurosci. 2004; 5: 85-96. <https://doi.org/10.1007/s10158-004-0031-y> PMID: 15480914

Coleman CM, Neckameyer WS. Serotonin synthesis by two distinct enzymes in *Drosophila melanogaster*. Arch Insect Biochem Physiol. 2005; 59: 12-31. <https://doi.org/10.1002/arch.20050> PMID: 15822093

Line 145-146: “The result showed that *PAH* and *PAHAL* mRNA were mainly expressed in cell bodies” – as opposed to what? The cell body is where all transcription and gene expression occurs (even if RNA is transported or localized elsewhere, it is still “expressed” from the nucleus/body). So I don’t know what the point of this sentence is, as all genes are expressed in the cell body (maybe the authors mean to say that the transcripts are localized only in the cell bodies).

Response:

We are sorry for the confusing concept of the cell bodies. In fact, the cell body is a fraction of neuron and contains the nucleus of neuron. A neuron can be divided into three domains: the dendrite, the cell body (also called soma or perikaryon), and the axon (Díaz-Nido et al., 2018; Hirokawa and Takemura, 2005). The locust brain is divided into two major regions: an outer cortical layer of cell bodies, and an inner neuropil area of fibers, e.g. mushroom bodies (Boyan et al., 1993). To avoid any misunderstanding, we revised the sentence as suggested to read: “The result showed that *PAH* and *PAHAL* transcripts were expressed in cell bodies of neurons. Furthermore, *PAHAL* colocalised with *PAH* in the locust brain (Fig 3C and S2 Fig)” (Lines 160 - 162). In addition, we have added a figure of Hochest 33342-stained nuclei to indicate the location of cell bodies of neurons (Fig S2, has been suppled in the revised manuscript).

Related references:

Díaz-Nido J, Hernández MA, Avila J. Microtubule proteins in neuronal cells. Microtubule Proteins: CRC Press; 2018. p. 193-257.

Hirokawa N, Takemura R. Molecular motors and mechanisms of directional transport in neurons. Nat Rev Neurosci. 2005; 6: 201-14. https://doi.org/10.1038/nrn1624 PMID: 15711600

Boyan G, Williams L, Meier T. Organization of the commissural fibers in the adult brain of the locust. J Comp Neurol. 1993; 332: 358-77. https://doi.org/10.1002/cne.903320308 PMID: 7687257

In Figure 2E I would prefer if the Y-axes in Figure 2 were not truncated – this makes the differences between the groups appear to be much larger than they actually are.

Response:

We’re happy to accept the reviewer’s suggestion, and present Fig. 2E with non-truncated Y-axes.

Figure 3B -- I do not understand why the "Rlative mRNA" (typo, should be "Relative") starts at 0.015, instead of 1. What is this mRNA being relativized to?
If the raw RNA-seq reads have been uploaded to a public database (e.g. Short Read Archive), this could be included (or perhaps it already here and I missed it).

Response:

Sorry for the oversight. We corrected to “Relative mRNA level” in Figure 3B. The expression levels in *PAHAL* and *PAH* were normalised against that of the internal control *rp49*. And we do not further normalize the expression data in this figure. Instead, we have normalized the relative expression from treatments against that from control in Fig. 2B, which has been dictated in figure legend (Line 972).

The RNA-seq raw data have been uploaded to NCBI with the accession number PRJNA522953. We’ll also upload the q-PCR data to Harvard Dataverse Network with the websit <https://dataverse.harvard.edu/dataset.xhtml?persistentId=doi:10.7910/DVN/LXRBKJ> (See line 679).

Grammatical suggestions:
Line 42: “mediated the transcription activation” –> “transcriptional”

Response:

Thanks for the reviewer’s suggestions. We have revised as suggested ( Line 44).

Line 57: “exhibits the spatiotemporal specific expression patterns” can delete “the”.

Response:

Done (Line 59)

Line 60: “regulate the gene expression” can delete “the”

Response:
Have revised as suggested (Line 62).

Line 66: "is" --> "are"

Response:

Done (Line 68).

# Reviewer #2:

Zhang et al., describe the neural expression a locust long non-coding RNA (lncRNA), *PAHAL*, and provide good evidence that it regulates expression of the gene encoding phenylalanine hydroxylase (PAH) by binding to the SRSF2 splicing factor and recruiting it to the *PAH* promoter. Evidence is presented that this regulatory mechanism controls dopamine (DA) amounts in the locust brain. The authors show that *PAHAL* is expressed in the brain (and in the nucleus) and that both it and *PAH* are elevated when locusts are in crowded conditions and decreased when the insects are isolated. This suggests a role for the lncRNA in the regulation of social behavior. Further, they show that RNAi-mediated reductions in *PAHAL* result in a transition from group to solitary behavior, supporting the idea that it can regulate such behavior. Additional studies indicate that *PAHAL* binds to a short segment of the proximal promoter region of *PAH*, providing a mechanism for regulation of the gene. Using immunoprecipitation assays and brain tissues, the authors show that *PAHAL* is bound to SRSF2, in vivo, and that SRSF2 amounts increase with locust crowding and decrease in solitary locusts, similar to what is observed for *PAHAL* and *PAH*. They use cell-based assays to show that SRSF2 enhances expression of *PAH* and that it interacts with *PAHAL* to regulate gene expression. Finally, the authors identify the sequences within *PAHAL* that bind SRSF2 for its recruitment to *PAH* as well as exonic-splicing enhancer (ESE) sites within *PAH* that mediate regulation by SRSF2. All of these results lead to a satisfying model in which *PAHAL* recruits SRSF2 to the *PAH* gene to regulate its expression, dopamine signaling and behavior.

I have no major issues with the manuscript, but the following should be addressed prior to publication:

-The Discussion section should be shortened considerably; it is far too long.

Response:
The discussion section has been shortened in the revised manuscript. We deleted the paragraph between old lines 276 - 291.

-What was the reason for thinking *PAHAL* regulated *PAH*. Was it just the proximity of *PAHAL* to *PAH* or was there another reason for believing that *PAHAL* might be regulatory? The authors may wish to state this explicitly.

Response:

We concluded the regulatory role of *PAHAL* because of at least three reasons:

1. The positional correlation, as mentioned by the reviewer. A novel lncRNA, *PAHAL* that overlaps with the *PAH* gene was indicated and verified by the data of the genome and transcriptomes of the locust and the experiments in Fig 1.

2. Expression correlation between *PAH* and *PAHAL*. The *PAHAL* knockdown reduced the *PAH* expression. Moreover, the RNA-seq data also showed that *PAH* was the most differentially expressed gene by *PAHAL* knockdown. However, *PAH* knockdown had no effect on *PAHAL* level. These results confirm, “LncRNA can accumulate to their sites of transcription and affect expression of neighboring genes (Chen, 2016).”

3.. *In vitro* validation. *PAHAL* might be regulatory is confirmed by subsequent luciferase assay.

We summarized these reasoning in Discussion (See lines 272 - 313).

Related reference:

Chen LL. Linking long noncoding RNA localization and function. Trends Biochem Sci. 2016; 41: 761-72. <https://doi.org/10.1016/j.tibs.2016.07.003> PMID: 27499234

-I was initially confused by the *PAHAL* knockdown experiment because it seemed like the dsRNA would target both *PAH* and *PAHAL*. I then realized that the dsRNA was targeted to an intron of *PAH*, so it ought to be specific for *PAHAL*. The authors should add a sentence in the appropriate section making this clear so that other readers will not be confused.

Response:

Thanks the reviewer for figuring out this confusing point. A sentence has been added to state: “The dsRNA and siRNAs of *PAHAL* were targeted to the right intron flanking the sixth exon in the *PAH* gene and ought to be specific for *PAHAL* not influence the pre-mRNA of *PAH*.” (Lines 123 – 124)

-p. 6, Please indicate somewhere in this first paragraph that primer sequences for making dsRNA and siRNAs can be found in supplemental materials.

Response:

We’d like to accept the reviewer’s suggestion and “Primer sequences of these dsRNAs and siRNAs can be found in table S3.” have been added (Line 125).

-p 8, it might be good here to remind the reader about G and S populations at the beginning of this section, even though they were described in the Intro. The average reader won't be as familiar with locust behavior as the authors.

Response:

We added a sentence at the beginning of this section to read: “G locusts display high mobility and sociable. By contrast, S locusts are sedentary and live at very low densities [41]” Line 154

-p 8, the images in Fig. 3C are difficult to interpret unless one happens to be a locust neurobiologist. Which cell bodies are shown? Are the RNAs expressed everywhere in the brain or is there a more specific expression pattern? Do the images represent the whole brain or part of the brain? Also, it looks like *PAHAL* is predominantly nuclear whereas *PAH* is cytoplasmic so I'm not sure if colocalization is the right word to use. I would say they are in the same cells with one being nuclear and the other cytoplasmic. Similarly, in Fig. 5B, what brain region is shown?

Response:

Thank you for your comments. We are sorry for the confusing concept of the cell bodies. In fact, the cell body that contains nucleus is the site of protein synthesis of neuron and the origination of dendrite (Díaz-Nido et al., 2018; Hirokawa and Takemura, 2005). The locust brain is divided into two major regions: an outer cortical layer of cell bodies, and an inner neuropil area of fibers, e.g. mushroom bodies (Boyan et al., 1993). To avoid any misunderstanding, we revised the sentence as suggested to read: “The result showed that *PAH* and *PAHAL* transcripts were expressed in cell bodies of neurons. Furthermore, *PAHAL* colocalised with *PAH* in the locust brain (Fig 3C and S2 Fig)” (Line 160 - 162).

In the revised manuscript, we also have added a Supplemental Fig. S1 to indicate outer cortical layer of cell bodiesof neurons in the locust brain by Hochest-stained nuclei (S2 Fig, has been done before the manuscript submission). The blue signal indicates the localisation of cell bodies in the locust brain. The merged signal of blue, red and green showed that *PAH* and *PAHAL* transcripts were mainly expressed in cell bodies compared with the Hochest (Fig 3C and S2 Fig).

These sentences have been added in figure legends of Fig 3C and Fig 5B.

1. “The "a" squares specifically indicate the brain region of the right images in the locust protocerebrum. The brain region of the Fig 5B is indicated by the "b" squares in the locust deutocerebrum”. (Lines 992 - 994)
2. “The brain region in these images was indicated by the "b" squares of Fig 3C”. (Line 1018)

Related references:

Díaz-Nido J, Hernández MA, Avila J. Microtubule proteins in neuronal cells. Microtubule Proteins: CRC Press; 2018. p. 193-257.

Hirokawa N, Takemura R. Molecular motors and mechanisms of directional transport in neurons. Nat Rev Neurosci. 2005; 6: 201-14. https://doi.org/10.1038/nrn1624 PMID: 15711600

Boyan G, Williams L, Meier T. Organization of the commissural fibers in the adult brain of the locust. J Comp Neurol. 1993; 332: 358-77. https://doi.org/10.1002/cne.903320308 PMID: 7687257

-p. 10, top, does siRNA or dsRNA for *PAH* and *PAHAL* have the same effects on behavior. Perhaps this is known from a previous publication. If so, please mention it.

Response:

We performed both dsRNA (Fig. 4B, D) and siRNA (Fig. 4C, E) knock down. The effects of the two types of RNA interference are not the same but similar, that is, *PAHAL* knock down by either dsRNA or siRNA injection drove behavioral transition from gregarious toward solitarious phase. To avoid any misunderstanding on these results, we added a sentence to read:

“We investigated the role of *PAHAL* in behavioural regulation in the locusts using dsRNA and siRNA interference of *PAHAL*, respectively.” (Line 172)

“We also examined the specific behavioural effect from siRNA knock down.” (Line 187)

-p. 11, bottom, please state clearly that SRSF2 was tagged with V5. The wording here is a little unclear.

Response:
This sentence “*In vitro* RIP using an antibody against V5 tag in SRSF2-V5 protein-overexpressed cell lysis revealed…” has been added in Line 227.

-p 13, there is reference to Fig 6C which I think should be 7C.

Response:
Error as “Fig 6C” has been corrected to “Fig 7C” in Line 264.

-Fig. 6 is missing the legend for panel F.

Response:

The legend for panel F of Fig 6 has been added in Line 1038.

-It's almost impossible to read the names of the genes shown in Fig 2C. Please create a larger figure for publication.

Response:

Fig 2 has been corrected.

-In some places *PAHAL* is referred to as mRNA. It’s not really a messenger RNA as it doesn’t encode a protein. It seems better to refer to it as RNA, not mRNA.

Response:

Such errors have been corrected, e.g. in Fig 3.

# Reviewer #3:

The authors investigate the regulation of the gene phenylalanine hydroxylase (PAH) in the locust, which plays a role in dopamine production in the brain that, in turn, regulates behavioral phase changes (solitary vs. gregarious). A model is proposed wherein a portion of the unspliced *PAH* transcript is separately transcribed as a sense long noncoding RNA (*PAHAL*) that positively regulates *PAH* transcription by recruiting a splicing factor protein that interacts with the *PAH* promoter. The regulatory potential of long noncoding RNAs is an exciting area of research. The authors present an impressive array of experimental data, including gene expression assays, RNA interference, RNA immunoprecipitation, mouse cell knockouts, and luciferase assays of *PAHAL* (and interacting factors) effects on transcription. The results of these experiments provide insights into *PAH* regulation important to dopamine-mediated locust phase transitions, but I have some concerns about the robustness of the described role of the long noncoding RNA, *PAHAL*, that is proposed to mediate neurotransmitter metabolism – and behavioral plasticity by orchestrating a local transcriptional loop.

Response:

We are very grateful to the reviewer for his or her valuable comments.

Could the *PAHAL* dsRNA used to target *PAHAL* also target *PAH* pre-mRNA (since both are in the nucleus) to directly decrease *PAH* expression?

Response:

The question that the reviewer asks is clearly an important one. It seems to be least possibility that the *PAHAL*-specific dsRNA also target *PAH* pre-mRNA to directly decrease *PAH* expression. We would like to address it from two aspects, in theory and with additional experiment. By its very biochemical nature, the pre-mRNA is processed in cascading events including 5' end capping, splicing out introns, and 3' end maturation by cleavage or polyadenylation while the nascent RNA chain is being synthesized by RNA polymerase II. Also, note that the physiological substrate for most processing factors is not a solitary pre-RNA but instead a ternary complex comprising a growing RNA chain, RNA polymerase II and mRNA processing factors (Zorio, 2004). Furthermore, Ago1-guided RNA degradation occurs at post-transcriptional level (Covarrubias and Reyes, 2010). Therefore, it has less chance that the intronic-specific dsRNA of *PAHAL* might also target quite a number of *PAH* pre-mRNA that has escaped the splicing and been exposed from the ternary complex. In fact, several previous work with intron-derived lncRNAs had used the same method of dsRNA or siRNA interfere to specifically knock down mRNA levels of the lncRNAs but not pre-mRNA of their local ancestral gene (e.g., Sun et al., 2016; Beckedorf et al., 2013). To further validate the result in our case, we added a “control” experiment to address the question. Specifically, we performed dsRNA knockdown targeting the first three exons of *PAH* which do not cover *PAHAL* by using primer 7 and 8 (Fig. 1B). The result shows that *PAH* knockdown significantly repressed *PAH* mRNA levels but had no effect on *PAHAL* levels. We have added the new data to the manuscript (Fig 2C, and see results in Line 130), and also the reference to Methods (Line 470).

Related references:

Zorio DAR, Bentley DL. The link between mRNA processing and transcription: communication works both ways. Exp Cell Res. 2004; 296: 91-7. <https://doi.org/10.1016/j.yexcr.2004.03.019> PMID: 15120999

Covarrubias AA, Reyes JL. Post-transcriptional gene regulation of salinity and drought responses by plant microRNAs. Plant Cell Environ. 2010; 33: 481-9. 10.1111/j.1365-3040.2009.02048.x PMID: 19781008

Beckedorff FC, Ayupe AC, Crocci-Souza R, Amaral MS, Nakaya HI, Soltys DT, et al. The intronic long noncoding RNA *ANRASSF1* recruits PRC2 to the *RASSF1A* promoter, reducing the expression of *RASSF1A* and increasing cell proliferation. PLoS Genet. 2013; 9: e1003705. <https://doi.org/10.1371/journal.pgen.1003705> PMID: 23990798

Sun TT, He J, Liang Q, Ren LL, Yan TT, Yu TC, et al. LncRNA *GClnc1* promotes gastric carcinogenesis and may act as a modular scaffold of WDR5 and KAT2A complexes to specify the histone modification pattern. Cancer Discov. 2016; 6: 784-801. <https://doi.org/10.1158/2159-8290.CD-15-0921> PMID: 27147598

Did the nuclear fractionation experiment differentiate *PAHAL* from *PAH* pre-mRNA?

Response:

This is a very good point. The nuclear fractionation experiment did not differentiate *PAHAL* from *PAH* pre-mRNA as they both are present in nucleus. However, the detection of both *PAHAL* and *PAH* pre-mRNA in nuclear fractionation using one RT-PCR does not compromise our conclusion about the nuclear retention of *PAHAL* because of two facts. First, pre-mRNA has much shorter half-life than mature RNA, and mostly are present transiently in the process of transcription (Iype et al., 2005). Secondly, pre-mRNA is generated in much lower abundance compared to their mature mRNA counterparts (Mukherjee et al., 2011; Iype et al., 2005). Therefore, we may have detected mainly *PAHAL* transcript in this experiment. In fact, other solid evidence provided by double fluorescence in situ hybridization (Fig. 3C) and nuclear location signal analysis (S3 Fig.) also confirm the nuclear retention of *PAHAL*. To present the experiment result more accurately, we revised the corresponding sentence to read: “The nuclear fractionation experiment showed that 94% of *PAHAL* mRNAs (possibly together with trace amount of *PAH* pre-mRNA) localises in the nucleus relative to nuclear RNA *U6* (positive control) and cytoskeleton actin (negative control).” (Line 196).

Related references:

Mukherjee N, Corcoran DL, Nusbaum JD, Reid DW, Georgiev S, Hafner M, et al. Integrative regulatory mapping indicates that the RNA-binding protein HuR couples pre-mRNA processing and mRNA stability. Mol Cell. 2011; 43: 327-39. 10.1016/j.molcel.2011.06.007 PMID: 21723170

Iype T, Francis J, Garmey JC, Schisler JC, Nesher R, Weir GC, et al. Mechanism of insulin gene regulation by the pancreatic transcription factor Pdx-1: application of pre-mRNA analysis and chromatin immunoprecipitation to assess formation of functional transcriptional complexes. J Biol Chem. 2005; 280: 16798-807. <https://doi.org/10.1074/jbc.M414381200> PMID: 15743769

For the luciferase assays, is it possible that the *PAHAL* enhancer effect is simply due to a genomic enhancer element residing in an intron? In other words, could it be that the effect on transcription is not typically RNA-mediated but is rather through DNA looping interactions with the promoter? Can the authors comment on whether/how their experiments preclude this possibility?

Response:

The point is well taken and we would love to provide a solid answer. Basically, the enhancer acts regardless their orientation (Serfling et al., 1985). Thus, to preclude the possibility of a genomic enhancer residing in *PAHAL*, we had designed a reverse *PAHAL*, and expressed *PAHAL* in the reverse orientation. The luciferase assay showed that *PAHAL*, but not reverse *PAHAL* substantially enhanced the luciferase expression driven by the *PAH* promoter in S2 cells (Fig 5C). This result indicates that *PAHAL* does not work as an enhancer sequence element. To make it clearer, we amended the result statement and discussion:

“By contrast, *PAHAL* expressed in the reverse orientation (i.e., reverse *PAHAL*) had no effect on *PAH* promoter activity (Fig 5C). Thus, *PAHAL* enhances the promoter activity of *PAH* not by acting as an enhancer element.” (Line 205 - 207)

“However, the activity assay with reverse *PAHAL* demonstrated that the enhancer effect rendered by *PAHAL* is not due to a genomic enhancer element residing in its sequence (Fig. 5C).” (Line 353 -355)

Related reference:

Serfling E, Jasin M, Schaffner W. Enhancers and eukaryotic gene transcription. Trends Genet. 1985; 1: 224-30. <https://doi.org/10.1016/0168-9525(85)90088-5>

Could the RNA pulldown with SRSF2 be capturing pre-mRNA in the nucleus that is being targeted by the spliceosome (including SRSF2, which is associated with nascent RNA and the spliceosome)?

Response:

We guess the reviewer is talking about the effect of RNA immunoprecipitation (RIP) but not RNA pulldown. If yes, we would like to keep our opinion on this count. While SRSF2 acts as a transcription activator in the present study, it is initially part of the transcription complex assembled at gene promoters. SRSF2 then starts RNA-dependent release from the complex and triggers transcription activation through binding to the promoter-associated nascent RNA (Ji et al., 2013). Based on this knowledge, we know that SRSF2 interacts with nascent RNA, but not pre-mRNA for transcription regulation. Meanwhile, the physical interaction between SRSF2 and *PAHAL* has been verified through RNA pulldown followed by mass spectrometry (Fig. 6A and Table S2) and western blot ((Fig. 6B). To make it clearer, we amended the statement: “To determine the physical interaction between *PAHAL* and SRSF2 that binds to the promoter-associated nascent RNA during transcription regulation, we conducted several *in vitro* and *in vivo* RNA immunoprecipitation (RIP).” (Lines 225 - 227)

Related reference:

Ji X, Zhou Y, Pandit S, Huang J, Li H, Lin CY, et al. SR proteins collaborate with *7SK* and promoter-associated nascent RNA to release paused polymerase. Cell. 2013; 153: 855-68. <https://doi.org/10.1016/j.cell.2013.04.028> PMID: 23663783

Line 81: I did not see PAH mentioned in the abstract or in a word search of ref 41. Can the link between microRNA-133 and *PAH* in this sentence be described more clearly? Was *PAH* knocked down in this prior study?

Response:
Sorry for the oversight. PAH is named as Henna in the locust in the ref. 41 (re-numbered as ref. 36 in the revised version of the manuscript, i.e., Yang et al., 2014) because *Drosophila* Henna functions as and is homology to vertebrate PAH (Ruiz-Vazquez and Silva, 1999). We now added the reference to the statement: “Phenylalanine hydroxylase (PAH, also known as Henna in *Drosophila* and *Locusta*) catalyses the synthesis of tyrosine from phenylalanine and … [34-37]”. (Lines 72 - 74).

Following the reviewer’s suggestion, we rewrote the sentence to read: “Specifically, miR-133 targeting *PAH* coding region repressed *PAH* expression, reduced DA production and drove locust soliterization while activated, and vice versa while inhibited [36].” (Line 85-87)

Yes, *PAH* was knocked down in a rescue experiment in this prior study. Specifically, *PAH* knockdown can rescue the effects caused by microRNA-133 inhibition that increased *PAH* expression and resulted in gregarious-like behaviors (Yang et al., 2014).

Related reference:

Yang M, Wei Y, Jiang F, Wang Y, Guo X, He J, et al. MicroRNA-133 inhibits behavioral aggregation by controlling dopamine synthesis in locusts. PLoS Genet. 2014; 10: e1004206. <https://doi.org/10.1371/journal.pgen.1004206> PMID: 24586212

Ruiz-Vazquez P, Silva FJ. Aberrant splicing of the *Drosophila melanogaster* phenylalanine hydroxylase pre-mRNA caused by the insertion of a *B104/roo* transposable element in the *Henna* locus. Insect Biochem Mol Biol. 1999; 29: 311-8. <https://doi.org/10.1016/S0965-1748(99)00002-8> PMID: 10333570

The senior author has argued that other single factors like a microRNA are responsible for modulating dopamine and behavioral plasticity in locusts in other publications. Can the authors take an integrative view in their discussion to describe how the findings in the present manuscript fit in with their prior findings about dopamine regulation in the locusts?

Response:

The point is well taken and we’re glad to provide an integrative view based on these findings. Our discussion is as follows: “Interestingly, two distinct regulatory mechanisms for *PAH* expression rendered by two noncoding RNAs have been employed in the locust. On one hand, lncRNA *PAHAL* is induced by locust crowding, positively regulates *PAH* expression and DA production in the brain at the transcriptional level. On the other hand, miR-133 is upregulated by locust isolation, evokes degradation of *PAH* mRNA and inhibits DA production, thus representing a mechanism for post-transcriptional regulation of *PAH* [36]. Both *PAHAL* and miR-133 are involved in the modulation of behavioral aggregation but in contrasting directions. Therefore, the two mechanisms at different regulatory levels may be orchestrated in response to population density to ensure the fine tuning of *PAH* expression and DA biosynthesis and thus guarantee the deliberate behavioral response of locusts.” (Lines 326 - 335)

Overall, I think it is an overstatement to conclude that “*PAHAL* modulates behavioral aggregation” as stated in the abstract and that “*PAHAL* mediates *PAH* transcriptional activation.” Some of my hesitance to accept these statements arises because it seems possible that *PAHAL* is produced only in response to *PAH* activation to further enhance transcription (what activates *PAHAL*?). I would like to see the authors respond to potential sources of uncertainty outlined above in my comments and more explicitly lay out the logic that counteracts such concerns or otherwise introduce more uncertainty to the interpretation of their findings where appropriate.

Response:

All the points raised by the reviewers are clearly very important. We have addressed all the issues with new data or evidences. We hope the reviewer feels satisfactory with our replies.

**Have all data underlying the figures and results presented in the manuscript been provided?**
Large-scale datasets should be made available via a public repository as described in the *PLOS Genetics* [data availability policy](http://journals.plos.org/plosgenetics/s/data-availability), and numerical data that underlies graphs or summary statistics should be provided in spreadsheet form as supporting information.
Reviewer #1: Yes
Reviewer #2: Yes
Reviewer #3: None

PLOS authors have the option to publish the peer review history of their article ([what does this mean?](https://journals.plos.org/plosgenetics/s/editorial-and-peer-review-process#loc-peer-review-history)). If published, this will include your full peer review and any attached files.

If you choose “no”, your identity will remain anonymous but your review may still be made public.

**Do you want your identity to be public for this peer review?** For information about this choice, including consent withdrawal, please see our [Privacy Policy](https://www.plos.org/privacy-policy).
Reviewer #1: No
Reviewer #2: No
Reviewer #3: No
