# Supplementary material for: Long noncoding RNA PAHAL modulates locust behavioural plasticity through the feedback regulation of dopamine biosynthesis
Source: PLoS Genet. 2020 Apr 29;16(4):e1008771. doi: 10.1371/journal.pgen.1008771 (PMC7241820; doi:10.1371/journal.pgen.1008771)
Supplement: Attachment — Submitted filename: Response_to_Reviewers_R2.docx [file pgen.1008771.s011.docx]

Dear Drs. Ewer and Copenhaver,

Thank you for handling the review of our manuscript entitled 'Long noncoding RNA *PAHAL* modulates locust behavioural plasticity through the feedback regulation of dopamine biosynthesis' (PGENETICS-D-20-00072R1). Below are our responses to the specific comments of the reviewer 2#:

We are glad to follow the reviewer’s suggestion, and revised the sentence as suggested to read: “The result showed that *PAH* and *PAHAL* transcripts are both localized to neuronal cell bodies, but as expected *PAH* is cytoplasmic whereas *PAHAL* is nuclear.”(Line 161). We modified the figure legends of Fig 3C and S2 Fig accordingly to read: “S2 Fig. Localisation of *PAHAL* and *PAH* in FISH.” (Line 697). “(C) Localisation of *PAHAL* and *PAH* in the G brains as revealed through double fluorescence in situ hybridization (FISH).” (Line 1006)

We agree with the reviewer’s opinion and have added a sentence to read: “Furthermore, *PAH* and *PAHAL* have broad expression patterns rather than being localized only in a subset of locust neurons.” (Line 163)

Following the reviewer’s suggestion, we pointed out the general location of the aminergic neurons in the locust brain relative to PAH and *PAHAL* in Discussion. “Immunocytochemical studies show that aminergic neurons present wide distribution in the locust brain [1-3] (Wendt and Homberg, 1992; Homberg, 1991; Homberg, 2002). Specifically, most dopaminergic neurons are in the upper division of the central body [2] (Wendt and Homberg, 1992). Serotoninrgic neurons distribute 6 groups that innervate the central complex (groups l-5) [1] (Homberg, 1991). Our study indicates that *PAH* and *PAHAL* are expressed in almost all neurons in the locust brain (Fig. 3C and 5B).” (Line 284 - 289)

Related references:

Homberg U. Neuroarchitecture of the central complex in the brain of the locust *Schistocerca gregaria* and *S. americana* as revealed by serotonin immunocytochemistry. J Comp Neurol. 1991; 303: 245-254. <https://doi.org/10.1002/cne.903030207> PMID: 2013639

Wendt B, Homberg U. Immunocytochemistry of dopamine in the brain of the locust *Schistocerca gregaria*. J Comp Neurol. 1992; 321: 387-403. <https://doi.org/10.1002/cne.903210307> PMID: 1506476

Homberg U. Neurotransmitters and neuropeptides in the brain of the locust. J Comp Neurol. 2002; 56: 189-209. <https://doi.org/10.1002/jemt.10024> PMID: 11810722

Please do not hesitate to let me know if you have any question about this paper. Thank you very much again for your attention to our paper．

Sincerely yours,

Le Kang, Dr., Professor,

Institute of Zoology, Chinese Academy of Sciences

Beichen West-road 1,

Beijing 100101

P.R. China

Tel: 86-10-64807399

E-mail: [lkang@ioz.ac.cn](mailto:lkang@ioz.ac.cn)
